# Supplementary material for: A global analysis of national cardiovascular disease control plans using a multi-agent artificial intelligence model
Source: PLOS Digit Health. 2026 Jun 1;5(6):e0001447. doi: 10.1371/journal.pdig.0001447 (PMC13225395; doi:10.1371/journal.pdig.0001447)
Supplement: S1 Text — (DOCX) [file pdig.0001447.s001.docx]

# **S1: Large Language Model Pipeline Architecture Overview**

We built a three-stage, multi-agent system to analyse National Cardiovascular Disease Control Plans (NCVDCPs) with granular, accurate results. The pipeline sequentially refines outputs and applies quality checks, enabling consistent assessment of heterogeneous policy documents that combine statistical data, clinical guidelines, and implementation plans in diverse formats.

## **Agent 1: OCR and Document Processing (Qwen2.5-VL-72B)**

The first agent uses Qwen2.5-VL-72B, an open-source 72-billion-parameter vision-language model optimized for complex document parsing and multimodal understanding. It delivers robust OCR and precise document-structure comprehension, representing a major advance in vision-language processing.

### **Technical Specifications and Capabilities**

Qwen2.5-VL uses transformer-based attention mechanisms specifically designed for document understanding, which is essential for processing academic and policy documents with complex layouts. On multilingual OCR benchmarks it achieved 92.3% accuracy on DocVQA (Document Visual Question Answering) and 89.7% on TextVQA (Text-based Visual Question Answering), confirming its effectiveness for complex document analysis. Its unique QwenVL HTML format enables comprehensive extraction of hierarchical NCVDCP content through several key innovations:

- **Spatial-Aware Text Extraction:** Precisely localises text with bounding-box coordinates and preserves spatial relationships (e.g., headers, subheaders, linked content blocks), maintaining layout-dependent meaning during digitization.
- **Multi-Column Layout Processing:** Maintains correct reading order across multi-column pages, so policy recommendations, statistical data, and implementation guidelines appear in the intended sequence - critical for NCVDCPs that present comparative data and parallel implementation strategies.
- **Integrated Visual Element Recognition:** Detects and contextualizes embedded figures, tables, charts, and diagrams using advanced computer-vision methods; identifies table structures, extracts tabular data, and preserves links between visuals and their textual descriptions/references.
- **Multilingual Processing: Supporting over 100 languages,** enabling analysis of NCVDCPs from diverse national contexts and healthcare systems - essential for global cardiovascular disease control efforts and cross-country comparative policy analysis.

### **Document Preprocessing Pipeline**

The Qwen2.5-VL system implements a comprehensive OCR preprocessing pipeline designed to handle the complexities of NCVDCP document formats:

- **Image Preprocessing and Noise Reduction:** Converts pages to grayscale to improve text–background contrast; applies Gaussian blurring and median filtering to address scanning irregularities and impulse noise; uses adaptive noise-reduction algorithms to correct document artifacts, including compression artifacts and age-related degradation; binarizes with Otsu’s thresholding to maximize separation of text from background.
- **Geometric Correction and Text Optimization:** Detects and corrects skew with Hough Transform deskewing to restore horizontal alignment; applies morphological dilation and erosion to refine character boundaries, connect fragmented glyphs, and remove residual noise - thereby optimizing images for LSTM-based recognition networks.
- **Text Detection and Segmentation:** Uses connected component analysis (CCA) to identify contiguous text regions; segments these into text blocks, lines, and words while preserving reading order; performs character segmentation via geometric analysis to isolate individual characters; recognizes characters with LSTM models that capture sequential dependencies and output per-character confidence scores.
- **Advanced Layout Recognition:** Identifies headers, footnotes, captions, and sidebar content and encodes their hierarchical relationships to preserve document structure; integrates LayoutLMv3 to analyse visual elements - tables, graphs, and diagrams - alongside text; employs Vision Transformers to process images holistically and capture fine-grained details such as chart annotations and data patterns.

## **Agent 2: Content Analysis and Structured Output Generation (Llama 4 Scout, 70B)**

The second agent uses Llama 4 Scout, an open-source 70-billion-parameter language model chosen for exceptional performance in policy analysis and legal reasoning. Comprehensive evaluations in 2025 showed superior capability to interpret complex policy documents and produce structured analytical outputs.

### **Model Performance and Selection Rationale**

The selection of Llama 4 Scout was based on rigorous benchmark evaluations that demonstrated its effectiveness in domains directly relevant to NCVDCP analysis:

- **Legal Reasoning Performance:** On LegalBench, Llama 4 Scout attains 0.75–0.82 accuracy across legal reasoning categories - relevant NCVDCPs that include regulatory language, compliance requirements, and implementation guidelines requiring sophisticated legal interpretation.
- **Policy Analysis Performance:** On PolicyQA, a policy-understanding benchmark, the model achieves an F1-score of 0.73, showing it can extract salient information from complex policy frameworks and produce coherent, multi-dimensional analyses.
- **Multidisciplinary Knowledge Integration:** The model records an overall MMLU score of 0.88, with strong results on MMLU-legal (jurisprudence and professional law), supporting analysis of NCVDCPs in the context of healthcare policy, legal frameworks, and international health regulations.
- **Policy Analysis Capabilities (beyond benchmarks):** It reliably understands policy structure, identifies key implementation strategies, and extracts quantitative targets and metrics - capabilities essential for systematic NCVDCP analysis.

### **Retrieval-Augmented Generation Architecture**

The analysis agent employs a sophisticated retrieval-augmented generation (RAG) framework that enhances its analytical capabilities through dynamic knowledge integration:

- **Framework-Specific Knowledge Base: The system maintains a comprehensive knowledge base containing detailed definitions and scoring criteria of framework elements.**

### **Structured Prompting and Output Generation**

The model employs structured prompting techniques designed to generate consistent, comprehensive analytical outputs:

- **Multi-Layered Prompt Architecture: The prompting system incorporates multiple layers of instruction, including task-specific guidelines, framework-based evaluation criteria, and output formatting requirements. This multi-layered approach ensures that the model** maintains focus on relevant analytical dimensions while producing outputs in the required JSON format.
- **JSON Output Structure: The system generates outputs** containing three primary sections:

1. **Response: A comprehensive analytical assessment of the** NCVDCP **section or element being evaluated, incorporating relevant framework** elements and providing detailed reasoning for conclusions drawn.
2. **Score:** Quantitative ratings based on predefined policy-comprehensiveness criteria.
3. **Score Reasoning:** Detailed justification for each score, identifying strengths and weaknesses, coverage of indicators, targets, and metrics, and specific, actionable recommendations for improvement where applicable.

## **Agent 3: Quality Assurance and Validation**

The third agent implements a comprehensive quality assurance framework using natural language processing metrics and validation techniques to ensure output reliability and consistency.

### **Multi-Dimensional Quality Assessment**

- **Readability Analysis:** Applies Flesch–Kincaid metrics to keep outputs accessible to policymakers, healthcare administrators, and implementation teams.
- **Semantic Coherence Measures:** Assesses logical consistency via cosine similarity between responses and sub-element definitions, enforcing a similarity range of 0.3 < cosine similarity < 0.8 to ensure responses are substantively related to framework definitions while maintaining sufficient analytical independence and avoiding mere restatement of definitional content.
- **JSON Schema Validation:** Rigorously validates all outputs against predefined JSON schemas, ensuring consistency across NCVDCP analyses and enabling systematic comparison and aggregation.
- **Completeness Verification:** Runs comprehensive checks to confirm that all required analytical components are present and sufficiently addressed in each output.

### **Advanced Quality Control**

- **Threshold-Based Re-analysis:** If any quality dimension falls below preset thresholds, the validation agent re-runs only the affected JSON sections. A composite quality score **<** 0.75 triggers re-analysis, with up to 3 iterations. The loop stops when all criteria meet acceptable standards or the iteration limit is reached.
- **Error Propagation Controls:** Implements detection and mitigation strategies to isolate faults and prevent cascading errors, ensuring problems in one analytical dimension do not degrade overall assessment quality.

## **System Integration and Workflow**

Coordinated sequential workflow (three agents)

- The agents run in sequence, each applying its strength at a distinct stage.
- Integrations presence information fidelity and enable progressive refinement and validation of analytical outputs throughout the process.
- End-to-end quality control is enforced at every stage.

**Cached Intermediate Outputs**

- The system caches outputs at each stage to ensure reproducible analysis and efficient re-processing when quality improvements are needed.
- Caching supports systematic quality auditing and maintains consistency across multiple analysis runs.

**Error Propagation Controls**

- Advanced error handling prevents issues in early stages from degrading downstream analyses.
- The system includes automatic rollback and alternative processing pathways when primary methods encounter difficulties.

Resulting advance

- This multi-agent architecture combines cutting-edge vision–language processing, sophisticated natural-language understanding, and robust quality assurance to deliver systematic, reliable analysis of complex policy documents at scale.

## **Technical Implementation and Infrastructure**

The comprehensive analysis of NCVDCPs was conducted using our three-agent system deployed on Amazon Web Services (AWS) Elastic Compute Cloud (EC2) infrastructure. We utilized a high-memory EC2 instance (r6i.8xlarge with 256 GB RAM and 32 vCPUs) to accommodate the requirements of running the 72-billion parameter Qwen2.5-VL model and 70-billion parameter Llama 4 Scout model with sufficient memory bandwidth for efficient inference.

### **Document Processing Pipeline**

NCVDCPs were processed through a standardized pipeline designed to handle the heterogeneous formats and languages of the 4545 national plans. Documents were uploaded directly to the EC2 instance via secure file transfer protocol (SFTP) using the instance's public IP address, enabling direct integration with the processing environment. The system supported multiple input formats including PDF, Microsoft Word documents, and scanned image files, with automatic format detection and routing to appropriate preprocessing modules.

The Qwen2.5-VL model processed documents page-by-page, maintaining spatial relationships between text blocks, tables, figures, and charts while generating structured outputs in JSON format that preserved document hierarchy and cross-references.

Following successful document parsing, the structured content was passed to the Analysis and Reasoning Agent powered by Llama 4 Scout. This agent systematically evaluated each of the 69 framework sub-elements by retrieving a JSON-structured version of the framework definitions and scoring criteria.

### **Real-Time Quality Control and Performance**

The Quality Assurance and Validation Agent performed real-time quality control through multiple validation layers. JSON schema validation ensured structural integrity of outputs, triggering automatic re-analysis when quality metrics fell below predetermined thresholds (composite quality score <0.75).

The EC2-hosted system processed individual NCVDCPs in an average of 60 minutes, representing a substantial efficiency gain over traditional manual analysis methods. Memory utilization peaked at approximately 180 GB during concurrent model execution, with CPU utilization averaging 78% during active processing. The system implemented automatic checkpointing every sub-element to ensure analysis continuity in case of computational interruptions.

### **Output Generation and Data Management**

Upon completion of analysis for each NCVDCP, the system generated standardized outputs including: (a) element-level and sub-element-level scores, (b) comprehensive narrative analyses for each framework component, and (c) detailed scoring rationales with specific textual evidence citations. All outputs were automatically uploaded to a secure S3 bucket with versioning enabled to maintain analysis provenance and enable quality auditing.

To ensure consistency across the 4545-country analysis, all NCVDCPs were processed using identical model configurations and framework parameters. The system maintained detailed logs of processing parameters, model responses, and validation metrics for each analysis session, enabling comprehensive reproducibility and quality assurance across the entire dataset.
