# Supplementary figures and images for: A global analysis of national cardiovascular disease control plans using a multi-agent artificial intelligence model
Source: PLOS Digit Health. 2026 Jun 1;5(6):e0001447. doi: 10.1371/journal.pdig.0001447 (PMC13225395; doi:10.1371/journal.pdig.0001447)

# **S2: Delphi Process Stage One Questionnaire Quantitative Scoring by Element**
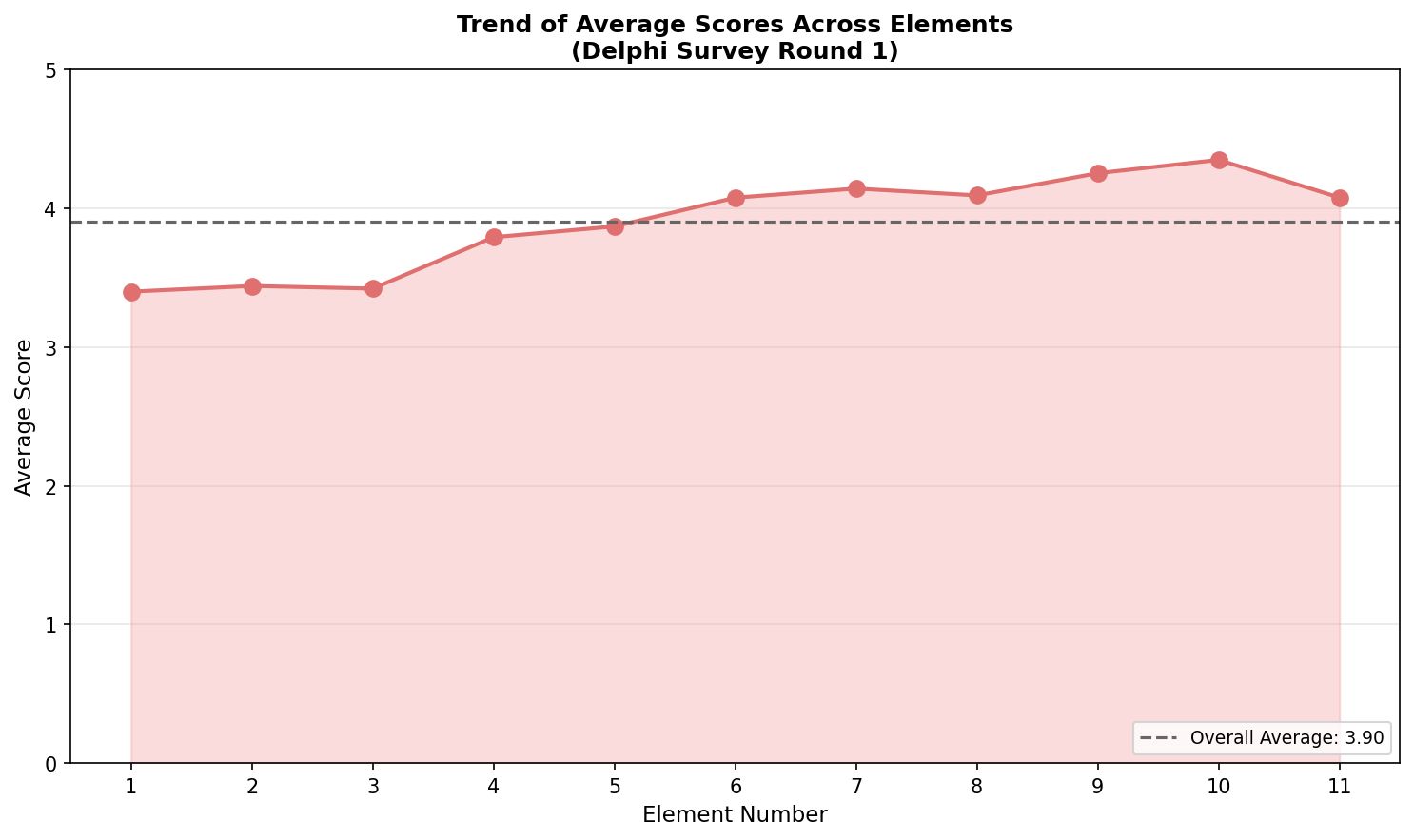


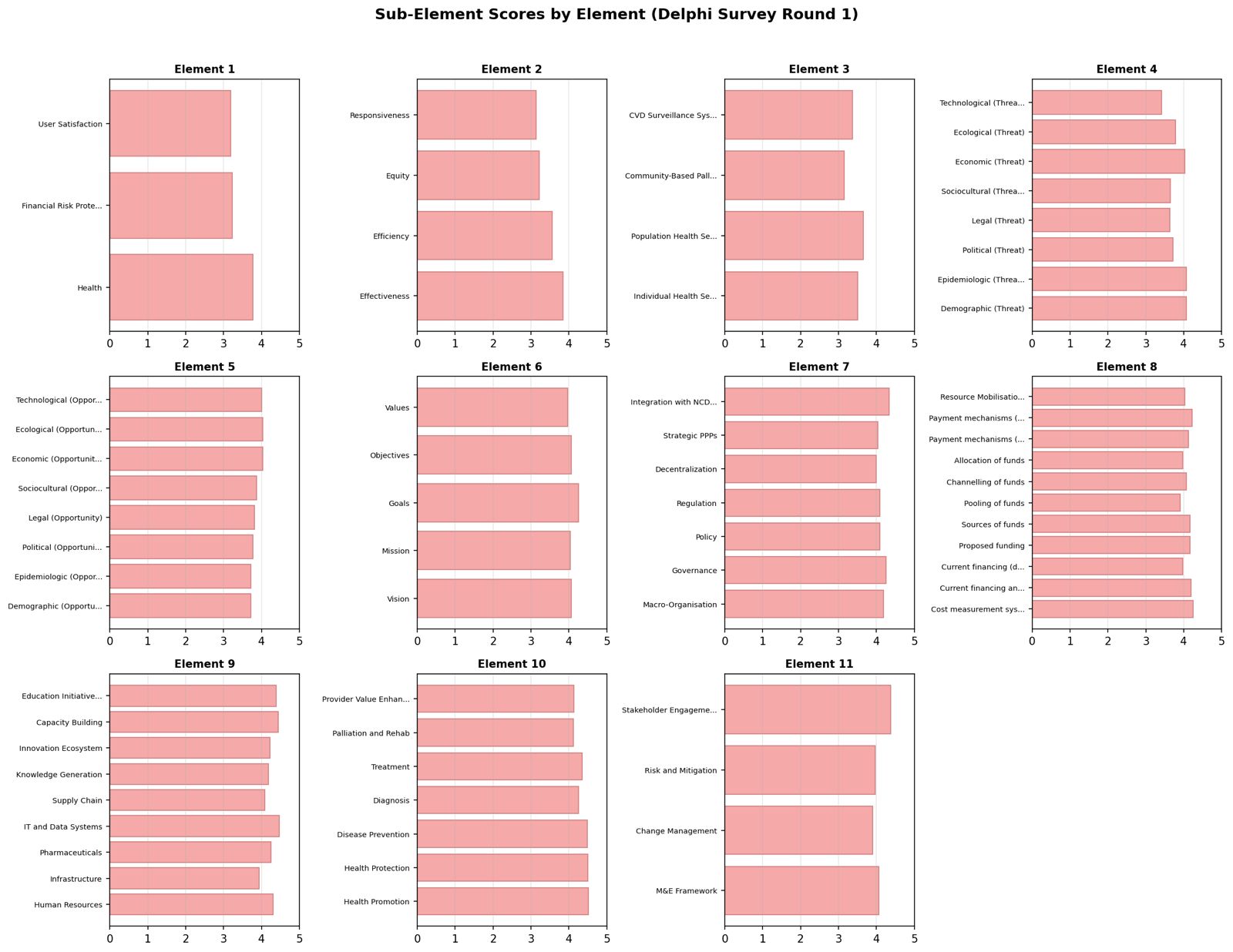

Supplement: S2 Text — (DOCX) [file pdig.0001447.s002.docx]
