# Supplementary material for: A global analysis of national cardiovascular disease control plans using a multi-agent artificial intelligence model
Source: PLOS Digit Health. 2026 Jun 1;5(6):e0001447. doi: 10.1371/journal.pdig.0001447 (PMC13225395; doi:10.1371/journal.pdig.0001447)
Supplement: S3 Text — (DOCX) [file pdig.0001447.s003.docx]

# **S3: Delphi Process Stage One Questionnaire Qualitative Feedback by Element**

| **Framework Element** | **Key Themes and Summary of Feedback** |
| --- | --- |
| 1. Health System Performance Outcomes | Holistic Metrics - Include wellbeing, quality of life, and patient satisfaction beyond clinical outcomes.  Data vs. Performance - Distinguish between data availability and actual system performance so countries aren't penalized just for lacking data.  Equity & Access - Explicitly track access (geographic, linguistic) and equity (urban vs. rural) separate from financial protection.  Oral Health - Integrate periodontal disease metrics (prevalence/treatment) given its link to CVD.  Specific Indicators - Recommended adding "MACE-free years," "Years with controlled risk factors," and "Premature CVD mortality." |
| 2. Health System Performance Objectives | Timeliness - Assess if care/prevention is delivered at the relevant time (early intervention).  Scope of Prevention - Acknowledge that many prevention factors (transport, food systems) lie outside the direct health system.  Resource Availability - Track the availability of resources (e.g., medicines, staff) distinct from patient access.  KPIs - Suggested tracking specific control rates (BP, HbA1c) and secondary prevention adherence. |
| 3. Health System Performance Outputs | Service Integration - Clarify the "right services" across all levels (primary to tertiary) and ensure public/private alignment.  Community Care - Expand "Community-based palliative care" to include integration with primary care and family support.  KPIs - Recommended specific metrics: screening rates, door-to-balloon times, 30-day readmission rates, and medication adherence.  Affordability - Strong call for free access to essential CVD medications. |
| 4. Health System Threats | Environmental Factors - Add noise and light pollution as environmental threats alongside air pollution.  External Economic Threats - Include global trade issues, tariffs, and taxes that affect medicine availability.  Aging & Awareness - Highlight the dual threat of rapidly aging populations and low public awareness of CVD risks.  Manufacturing - Lack of local manufacturing capacity for essential medicines is a key supply threat. |
| 5. Health System Opportunities | Digital Reach - Leverage high mobile phone penetration for public awareness campaigns.  Public Health Intersection - Better integrate clinical care with public health infrastructure.  Oral-Systemic Integration - Use the link between oral health and CVD as an opportunity for joint prevention programs.  Political Will - Address the challenge of weak political commitment to NCD taxes/regulations in LMICs. |
| 6. CVD Strategy | Political Durability - Strategies must be robust enough to withstand electoral cycles and changes in government.  Equity Targets - Goals should explicitly include targets for reducing disparities, not just improving aggregate averages.  Operational Focus - Objectives must bridge the gap between high-level policy and actual practice (e.g., workforce training). |
| 7. Governance & Organization | Integration - Emphasize shared data infrastructure and unified NCD frameworks rather than siloed CVD programs.  KPIs - Track the percentage of patients managed through coordinated/multidisciplinary programs (e.g., diabetes + CVD). |
| 8. Financing | Value-Based Financing - Shift from volume-based to value-based financing models.  Infrastructure Investment - Ensure financing covers public health infrastructure, not just clinical services.  Outcome Linking - Financing metrics should link spending directly to health outcomes (e.g., prevention funding $\to$ hypertension control). |
| 9. Resource Management | Workforce - Focus on task-shifting, multidisciplinary teams, and continuous capacity building/training.  Low-Cost Interventions - Prioritize simple, scalable interventions like low-sodium salts and polypills.  Digital Systems - Use digital tools for real-time monitoring and resource allocation.  Oral Health Workforce - Integrate dental professionals into the CVD care team. |
| 10. Health Services | Prevention Emphasis - Prioritize prevention and healthy behaviors (e.g., AHA's Life's Essential 8).  Oral Health Screening - Include periodontal assessments in CVD screening protocols.  Innovation - Track the uptake of new technologies and the Health Technology Assessment (HTA) process. |
| 11. Implementation | Inequalities - Explicitly monitor outcome inequalities.  Feedback Loops - Ensure M&E findings directly inform updates to clinical guidelines and policy.  Stakeholder Engagement - Establish permanent multi-sectoral committees that include patient representatives. |
| Cross-Cutting Feedback | Vulnerable Populations - Explicitly mention inclusivity for vulnerable groups throughout the framework.  Feasibility - Implementation feasibility should be a key indicator of success.  Oral Health - A strong, recurring theme was the need to integrate oral health (periodontal disease) as a strategic axis of the framework.  WHO Alignment: Ensure full alignment with WHO Global Action Plans and HEARTS technical packages. |
