# Supplementary material for: A global analysis of national cardiovascular disease control plans using a multi-agent artificial intelligence model
Source: PLOS Digit Health. 2026 Jun 1;5(6):e0001447. doi: 10.1371/journal.pdig.0001447 (PMC13225395; doi:10.1371/journal.pdig.0001447)
Supplement: S4 Text — (DOCX) [file pdig.0001447.s004.docx]

# **S4: Delphi Process Stage Two Qualitative Feedback Summary by Key Themes**

| **Key Theme** | **Expert Feedback Summary Improvements** | **Integration into Framework** |
| --- | --- | --- |
| Equity, Gender and Inclusivity | Participants emphasized the need to explicitly factor in gender disparities in trials and treatment. There was also a specific call to include pregnancy-related cardiovascular diagnoses. | Element 2 (Equity): Added specific references to gender disparities.  Element 1 (Outcomes): Included pregnancy-related complications in health outcome metrics. |
| Economic Value & Human Capital | The panel argued that health targets alone are insufficient; the framework should track human capital outcomes (productivity, life years saved) and economic metrics (affordability, utilization) to demonstrate value. | Element 1 (Outcomes): Added metrics for financial risk protection.  Element 8 (Financing): Reinforced economic evaluation metrics. |
| Operational Realism & Context | Experts challenged the framework to differentiate expectations for countries with varying development levels and emphasized that "availability" of diagnostics is useless without trained personnel to use them. | Element 6 (Strategy): Adjusted to allow for more flexible interpretation based on national context.  Element 9 (Resources): expanded metrics to include personnel training/capacity, not just equipment. |
| Modernizing Care Delivery | Feedback highlighted the shift away from clinic-centric models. Experts called for a distinction between clinic and community-based programs and the inclusion of digital innovations (virtual rehab, new tech integration). | Element 3 (Outputs): Differentiated community-based management from clinic-based care.  Element 9 (Resources): Added metrics for technology integration pathways. |
| Clinical Precision | Specific clinical gaps were identified, particularly regarding obesity management (as a distinct target), hypertension control rates, and CVD-specific mortality. | Element 1 & 3: Added specific indicators for total CVD mortality, obesity treatment coverage, and hypertension control. |
| System Governance | Suggestions included tracking transportation regulations (as a social determinant) and using transparency/corruption indices to measure governance. | Element 7 & 10: Added indicators for transportation policy and governance transparency. |
