# Supplementary material for: A global analysis of national cardiovascular disease control plans using a multi-agent artificial intelligence model
Source: PLOS Digit Health. 2026 Jun 1;5(6):e0001447. doi: 10.1371/journal.pdig.0001447 (PMC13225395; doi:10.1371/journal.pdig.0001447)
Supplement: S5 Text — (DOCX) [file pdig.0001447.s005.docx]

# **S5: Quantitative Analysis of National Cardiovascular Disease Control Plans across all sub-elements by median and score**

# **Element 1: Current health system performance for CVD control in relation to outcomes**

With a median of 0.67 [IQR: 0.33, 0.75], health system performance outcomes were minimally comprehensive across the 45 countries analysed. No country achieved full linkage of all three outcome factors to system inputs and outputs (score 5).

## **Health sub-element**

65.9% (29/45) of countries scored 1, indicating their CVD plans discuss some health outcomes but do not use specific indicators such as CVD-specific mortality rates, DALYs lost due to CVD, or 5-year survival rates for acute MI to assess health system performance. According to the framework, this represents basic acknowledgment without systematic measurement. 22.7% (10/45) scored 0, providing no discussion of health outcomes at all. 6.8% (3/45) scored 2, demonstrating both discussion and some indicator use. Only 2.3% (1/45) each achieved scores of 3 (comprehensive indicators) and 4 (with baseline data). No countries achieved score 5 (full linkage with performance targets). Median score: 1.0, reflecting limited engagement with health outcome measurement.

## **Financial Risk Protection sub-element**

56.8% (25/45) of countries scored 1, indicating they discuss some outcomes related to financial risk protection but lack recommended indicators such as out-of-pocket payments as a percentage of total health expenditure or incidence of catastrophic health expenditure for CVD care. This score represents conceptual acknowledgment without systematic measurement. 29.5% (13/45) scored 0, providing no discussion of financial protection. 9.1% (4/45) scored 2, showing both discussion and some indicator use. 4.5% (2/45) scored 3 with comprehensive indicators. No countries achieved scores 4 or 5. Median score: 1.0, suggesting most countries acknowledge CVD's financial burden but lack comprehensive protection frameworks.

## **User Satisfaction sub-element**

65.9% (29/45) of countries scored 0, indicating no discussion of user satisfaction outcomes such as patient-reported experience measures (PREMs) for CVD care, measures of shared decision-making in treatment choices, or patient trust in health providers. This high proportion at score 0 represents the weakest performance across all outcome sub-elements. 9.1% (4/45) scored 1, meaning they discuss some user satisfaction outcomes without specific indicators. Only 2.3% (1/45) scored 2 with some indicator use. No countries achieved scores 3, 4, or 5. Median score: 0.0, indicating user satisfaction receives minimal systematic attention in CVD planning.

# **Element 2: Current health system performance for CVD control in relation to objectives**

With a median of 0.50 [IQR: 0.25, 1.00], health system performance objectives showed limited comprehensiveness across the 45 countries. Effectiveness showed slightly better performance, but equity and responsiveness significantly lagged. No country fully integrated all four objectives with performance targets and outcome linkages.

## **Effectiveness sub-element**

50.0% (22/45) of countries scored 1, discussing health service effectiveness in relation to CVD but without specific indicators such as percentage of hypertensive patients with controlled blood pressure or proportion of eligible patients prescribed appropriate secondary prevention medications. This represents basic conceptual discussion without measurement systems. 25.0% (11/45) scored 0, providing no discussion of effectiveness. 11.4% (5/45) scored 2 with some indicator use. Notably, 13.6% (6/45) achieved score 4 with comprehensive indicators and baseline data. No countries scored 3 or 5. Median score: 1.0, indicating basic engagement with effectiveness measurement.

## **Efficiency sub-element**

40.9% (18/45) of countries scored 0, indicating no discussion of CVD service efficiency or use of indicators such as average length of stay for CVD-related hospitalizations, cost per CVD-related hospitalization, or health expenditure as a percentage of GDP. This high proportion at score 0 indicates efficiency remains underprioritized in CVD planning. 38.6% (17/45) scored 1 with basic discussion. 11.4% (5/45) scored 2, 2.3% (1/45) scored 3, and 6.8% (3/45) achieved score 4. No countries achieved score 5. Median score: 1.0, highlighting significant gaps in efficiency assessment.

## **Equity sub-element**

65.9% (29/45) of countries scored 0, indicating no discussion of health service equity for CVD or use of indicators such as disparities in CVD mortality rates by socioeconomic status, differences in control rates by ethnicity, or gender gaps in time from symptom onset to treatment for acute MI. This represents markedly weaker performance compared to other objectives. 31.8% (14/45) scored 1 with basic discussion but no disaggregated data. Only 2.3% (1/45) scored 2 with some indicators. No countries achieved scores 3, 4, or 5. Median score: 0.0, demonstrating a critical gap in equity-focused CVD planning.

## **Responsiveness sub-element**

59.1% (26/45) of countries scored 0, showing no discussion of health system responsiveness for CVD patients or indicators such as patient satisfaction scores with CVD care, average wait times for cardiology appointments, or percentage of patients reporting good communication with their provider. 31.8% (14/45) scored 1 with basic discussion. 2.3% (1/45) scored 3, and 6.8% (3/45) achieved score 4 with baseline data. No countries scored 2 or 5. Median score: 0.0, suggesting responsiveness to patient needs is largely overlooked in CVD planning.

# **Element 3: Current health system performance for CVD control in relation to outputs**

With a median of 0.75 [IQR: 0.50, 1.00], health system performance outputs showed limited comprehensiveness across all sub-elements. No country fully linked output factors to objectives and inputs with comprehensive targets.

## **Individual Health Services sub-element**

65.9% (29/45) of countries scored 1, discussing some individual health services for CVD but without specific indicators to measure output such as availability of essential CVD diagnostics at primary care level, proportion of primary care facilities offering CVD risk assessment, or availability of cardiac rehabilitation programs. According to the framework, this represents basic acknowledgment of services without measurement. 20.5% (9/45) scored 0, providing no discussion. 11.4% (5/45) scored 2 with some indicators. 2.3% (1/45) scored 3 with comprehensive indicators. No countries achieved scores 4 or 5. Median score: 1.0, indicating basic but incomplete service planning.

## **Population Health Services sub-element**

56.8% (25/45) of countries scored 1, discussing some population health services for CVD prevention without comprehensive indicators such as coverage rate of national smoking cessation programs, proportion of schools with healthy food policies, or coverage of salt reduction initiatives. 34.1% (15/45) scored 0, providing no discussion. 6.8% (3/45) scored 2, and 2.3% (1/45) scored 3. No countries achieved scores 4 or 5. Median score: 1.0, suggesting population-level service planning remains underdeveloped.

## **Community-Based Palliative Care sub-element**

97.7% (43/45) of countries scored 0, indicating no discussion of community-based palliative care services for advanced CVD patients such as home-based palliative care, availability of oral morphine in community health settings, or trained community health workers providing palliative care. This near-universal absence represents the weakest performance across all output sub-elements. Only 2.3% (1/45) scored 1 with basic mention. No countries achieved scores 2 through 5. Median score: 0.0, highlighting a critical gap in end-of-life care planning for CVD.

## **CVD Surveillance Systems sub-element**

63.6% (28/45) of countries scored 1, mentioning CVD surveillance systems but without comprehensive indicators such as existence of a national population-based CVD registry, completeness of vital registration data for CVD mortality, or frequency of national risk factor surveys. According to the framework, this represents basic acknowledgment without systematic implementation. 20.5% (9/45) scored 0, providing no discussion. 4.5% (2/45) scored 2, and 11.4% (5/45) achieved score 3 with comprehensive indicators. No countries achieved scores 4 or 5. Median score: 1.0, reflecting limited surveillance infrastructure development.

# **Element 4: Contextual threats shaping the health system CVD control response**

With a median of 0.12 [IQR: 0.12, 0.29], contextual threats were the least comprehensive element across all CVD plans from the 45 countries. No country analysed threat factors with recommended indicators and threat characterization in terms of certainty, magnitude, and timing.

## **Demographic Threats sub-element**

93.2% (41/45) of countries scored 0, indicating no discussion of demographic threats such as aging populations, dependency ratios, or urban versus rural population distribution that could affect CVD burden. This near-universal absence shows demographic threats are almost completely overlooked in CVD planning. Only 2.3% (1/45) each scored 1, 2, and 4. No countries scored 3 or 5. Median score: 0.0.

## **Epidemiologic Threats sub-element**

47.7% (21/45) of countries scored 1, discussing some epidemiologic threats without comprehensive indicators such as prevalence of hypertension, obesity, or tobacco use. This represents the strongest threat assessment within this element, though still limited. 22.7% (10/45) scored 0, providing no discussion. 18.2% (8/45) scored 2, 6.8% (3/45) scored 3, and 4.5% (2/45) achieved score 4 with comprehensive quantification. No countries achieved score 5 with full analysis of certainty, magnitude, and timing. Median score: 1.0.

## **Political Threats sub-element**

84.1% (37/45) of countries scored 0, showing no discussion of political threats such as lack of political stability, governance effectiveness, or absence of national NCD/CVD action plans. 9.1% (4/45) scored 1, and 2.3% (1/45) scored 2. No countries achieved scores 3, 4, or 5. Median score: 0.0, indicating political threats are rarely considered in CVD planning.

## **Legal Threats sub-element**

88.6% (39/45) of countries scored 0, indicating no discussion of legal threats such as absence of regulations on food labeling, restrictions on advertising unhealthy foods to children, or weak tobacco control legislation. 6.8% (3/45) scored 1 with basic discussion. Notably, 4.5% (2/45) achieved score 5, providing comprehensive threat analysis with certainty, magnitude, and timing assessments. No countries scored 2, 3, or 4. Median score: 0.0, showing legal frameworks are largely absent from CVD threat assessments.

## **Sociocultural Threats sub-element**

95.5% (42/45) of countries scored 0, indicating no discussion of sociocultural threats such as low health literacy, high tobacco use prevalence, cultural dietary patterns, or structural biases leading to CVD care disparities. Only 4.5% (2/45) scored 2. No countries scored 1, 3, 4, or 5. Median score: 0.0, representing near-complete absence of sociocultural threat analysis.

## **Economic Threats sub-element**

84.1% (37/45) of countries scored 0, showing no discussion of economic threats such as poverty rates, income inequality (GINI coefficient), low affordability of essential CVD medications, or economic instability affecting health system funding. 9.1% (4/45) scored 1, and 4.5% (2/45) scored 2. No countries achieved scores 3, 4, or 5. Median score: 0.0, highlighting limited consideration of economic barriers to CVD control.

## **Ecological Threats sub-element**

95.5% (42/45) of countries scored 0, indicating no discussion of ecological threats such as air pollution (PM2.5 levels), urban environments unsupportive of healthy lifestyles, limited green space access, or climate change impacts on vulnerable populations with CVD. Only 4.5% (2/45) scored 1. No countries achieved scores 2 through 5. Median score: 0.0, showing environmental determinants of CVD are almost completely overlooked.

## **Technological Threats sub-element**

97.7% (43/45) of countries scored 0, indicating no discussion of technological threats such as the digital divide limiting access to digital health tools, lack of health information system interoperability, or potential for health misinformation related to CVD prevention. Only 2.3% (1/45) scored 2. No countries scored 1, 3, 4, or 5. Median score: 0.0, representing the weakest threat assessment across all sub-elements.

# **Element 5: Contextual opportunities shaping the health system CVD control response**

With a median of 0.29 [IQR: 0.12, 0.50], contextual opportunities were among the least comprehensive elements across the 45 countries. No country systematically analysed all opportunity factors with comprehensive indicator use and opportunity characterization.

## **Demographic Opportunities sub-element**

63.6% (28/45) of countries scored 0, indicating no discussion of demographic opportunities such as favorable dependency ratios, large youth populations allowing for early CVD prevention, or urbanization that could centralize specialized services. 31.8% (14/45) scored 1 with basic discussion. 4.5% (2/45) scored 2. No countries achieved scores 3, 4, or 5. Median score: 0.0, suggesting demographic opportunities for CVD prevention are widely missed.

## **Epidemiologic Opportunities sub-element**

The distribution was notably even across the lower scores: 31.8% (14/45) of countries each scored 0, 1, and 2. This relatively stronger and more varied engagement reflects opportunities such as high population awareness of CVD risk factors, existing programs for integrated prevention, or high rates of influenza vaccination that can be leveraged for opportunistic CVD prevention messaging. 2.3% (1/45) each scored 3 and 4. No countries achieved score 5. Median score: 1.0, representing the strongest opportunity assessment within this element.

## **Political Opportunities sub-element**

50.0% (22/45) of countries scored 0, indicating no discussion of political opportunities such as strong government stability, high political will to address NCDs, alignment with key opinion leaders, or commitment to investing in primary healthcare and prevention. 22.7% (10/45) scored 1, and 6.8% (3/45) scored 2. No countries achieved scores 3, 4, or 5. Median score: 0.0, showing political opportunity identification remains limited.

## **Legal Opportunities sub-element**

70.5% (31/45) of countries scored 0, showing no discussion of legal opportunities such as existing strong regulatory frameworks that enable public health action, promote healthy environments, or ensure access to care. 6.8% (3/45) scored 1. No countries achieved scores 2 through 5. Median score: 0.0, indicating legal opportunities are largely unexplored.

## **Sociocultural Opportunities sub-element**

90.9% (40/45) of countries scored 0, indicating no discussion of sociocultural opportunities such as improving health literacy, cultural shifts toward healthier lifestyles, or community-based health promotion traditions. 4.5% (2/45) each scored 1 and 2. No countries achieved scores 3, 4, or 5. Median score: 0.0, showing sociocultural opportunities remain unexplored.

## **Economic Opportunities sub-element**

75.0% (33/45) of countries scored 0, showing no discussion of economic opportunities such as potential productivity gains from CVD treatment, opportunities for local pharmaceutical manufacturing, or economic growth enabling greater health investment. 13.6% (6/45) scored 1, and 2.3% (1/45) scored 2. No countries achieved scores 3, 4, or 5. Median score: 0.0, highlighting limited economic opportunity analysis.

## **Ecological Opportunities sub-element**

88.6% (39/45) of countries scored 0, indicating no discussion of ecological opportunities such as urban planning for walkability, green space access for physical activity, or clean energy transitions reducing air pollution. 6.8% (3/45) scored 1, and 4.5% (2/45) scored 2. No countries achieved scores 3, 4, or 5. Median score: 0.0, showing environmental opportunities are largely overlooked.

## **Technological Opportunities sub-element**

56.8% (25/45) of countries scored 0, indicating no discussion of technological opportunities such as telemedicine for CVD management, mobile health applications for risk factor monitoring, or digital platforms for cardiac rehabilitation. 40.9% (18/45) scored 1 with basic discussion. 2.3% (1/45) scored 2. No countries achieved scores 3, 4, or 5. Median score: 0.0, though this represents relatively stronger performance within opportunities, suggesting growing but limited recognition of technological potential.

# **Element 6: Strategy for CVD control**

With a median of 2.80 [IQR: 2.20, 3.20], CVD strategy elements were the most comprehensive across all elements from the 45 countries, demonstrating strong strategic planning foundations. Vision, goals, objectives, and values showed particularly strong performance.

## **Vision sub-element**

40.9% (18/45) of countries scored 4, indicating their plans provide forward-looking vision statements that are descriptive of long-term goals for CVD control with documented high-level political endorsement signifying commitment. This represents strong visionary leadership. 36.4% (16/45) achieved score 5, explicitly aligning their vision with relevant international targets and strengthening primary healthcare. 20.5% (9/45) scored 0, providing no vision statement. 2.3% (1/45) scored 3. No countries scored 1 or 2. Median score: 4.0, representing the strongest performance across all sub-elements and demonstrating robust visionary foundations in CVD planning.

## **Mission sub-element**

36.4% (16/45) of countries scored 1, indicating their mission statements are provided but unclear or do not define the plan's core purpose for CVD control. 31.8% (14/45) scored 2, defining a purpose but not specifying roles for key stakeholders or lacking clear governance structure. 29.5% (13/45) scored 0, providing no mission statement. Only 2.3% (1/45) achieved score 4 with clear purpose, stakeholder roles, and defined governance structure. No countries scored 3 or 5. Median score: 1.0, showing most plans establish basic mission statements but lack comprehensive stakeholder and governance articulation.

## **Goals sub-element**

77.3% (34/45) of countries scored 3, indicating their goals are aligned with the vision/mission and have some SMART (Specific, Measurable, Achievable, Relevant, Time-bound) targets, but these do not comprehensively cover all CVD outcome domains such as health outcomes, financial risk protection, and user satisfaction. This high concentration at score 3 demonstrates strong goal-setting capabilities globally. 11.4% (5/45) scored 2, 4.5% (2/45) each scored 0 and 4, and 2.3% (1/45) scored 1. No countries achieved score 5 with equity-focused targets. Median score: 3.0.

## **Objectives sub-element**

68.2% (30/45) of countries scored 3, indicating their objectives are linked to goals and have some SMART targets, but do not comprehensively cover all performance dimensions of efficiency, effectiveness, equity, and responsiveness. 15.9% (7/45) each scored 0 and 4. No countries scored 1, 2, or 5. Median score: 3.0, showing effective objective articulation in most CVD plans.

## **Values sub-element**

68.2% (30/45) of countries scored 4, indicating their core values are defined and linked to principles of CVD prevention and management with descriptions of how these values guide stakeholder behaviour and decision-making. This represents strong values articulation. 22.7% (10/45) scored 0, providing no values. 4.5% (2/45) each scored 1 (values mentioned but not defined) and 5 (values connected to ethical oversight mechanisms). No countries scored 2 or 3. Median score: 4.0, suggesting values are strongly articulated in most CVD plans with clear behavioral guidance.

# **Element 7: Proposed governance and organization interventions to enhance CVD control**

With a median of 2.14 [IQR: 1.43, 2.43], governance and organization interventions showed moderate comprehensiveness across the 45 countries. Governance and regulation sub-elements performed strongly, while strategic public-private partnerships and NCD integration showed significant gaps.

## **Macro-Organisation sub-element**

22.7% (10/45) of countries each scored 1 and 4, creating a bimodal distribution. Among those scoring 4, plans designate a lead agency with detailed responsibilities and inter-agency coordination mechanisms. 20.5% (9/45) achieved score 5, providing complete governance organograms with clear accountability structures. 18.2% (8/45) scored 0, 9.1% (4/45) scored 2, and 6.8% (3/45) scored 3. Median score: 3.0, showing variable but generally adequate organizational planning.

## **Governance sub-element**

47.7% (21/45) of countries scored 4, describing how greater governance will be promoted in all four critical areas: accountability and transparency, multi-sectoral collaboration, citizen engagement, and corruption prevention. This represents strong governance framework development. 40.9% (18/45) achieved score 5, providing all four governance areas with specific practical steps and responsible parties for each. 11.4% (5/45) scored 0. No countries scored 1, 2, or 3. Median score: 4.0, representing strong governance planning globally.

## **Policy sub-element**

47.7% (21/45) of countries scored 0, indicating no listing or description of relevant domestic or global policies influencing the CVD plan. 25.0% (11/45) scored 3, listing policies with some implications described. 11.4% (5/45) scored 1, 6.8% (3/45) scored 2, 4.5% (2/45) scored 4, and 2.3% (1/45) achieved score 5 with comprehensive policy analysis. Median score: 1.0, showing policy integration remains underdeveloped in many plans.

## **Regulation sub-element**

75.0% (33/45) of countries scored 4, describing new or strengthened regulations affecting four of the five critical areas: licensing and accreditation, quality assurance with clinical guidelines, public-private provider relationships, health technology assessment, and procurement. This represents one of the strongest performances across all sub-elements. 18.2% (8/45) scored 0, 4.5% (2/45) achieved score 5 covering all five areas, and 2.3% (1/45) scored 3. No countries scored 1 or 2. Median score: 4.0, demonstrating strong regulatory planning across CVD plans.

## **Decentralisation sub-element**

43.2% (19/45) of countries scored 4, providing comprehensive decentralization plans with capacity-building approaches for sub-national implementation specifically to strengthen primary and community-level care. 29.5% (13/45) scored 0, and 27.3% (12/45) scored 3 with some capacity-building approaches. No countries scored 1, 2, or 5. Median score: 3.0, indicating moderate attention to decentralization with clear room for improvement.

## **Strategic Public-Private Partnerships sub-element**

72.7% (32/45) of countries scored 0, indicating no discussion of strategic public-private partnerships for CVD control such as PPPs for infrastructure development, service delivery, or pharmaceutical supply. This high proportion at score 0 represents the weakest governance sub-element. 13.6% (6/45) scored 1, 6.8% (3/45) scored 2, and 2.3% (1/45) each scored 3, 4, and 5. Median score: 0.0, highlighting a significant gap in partnership strategy development.

## **Integration with Noncommunicable Disease Programs sub-element**

Data for this sub-element was limited. Most plans did not explicitly address integration mechanisms with other NCD programs such as diabetes, cancer, and chronic respiratory disease control initiatives. According to the framework, score 0 indicates no discussion of CVD-NCD integration, while score 5 represents comprehensive integration with shared risk factors, resources, and performance targets linked to improved CVD and NCD outcomes. Where assessed, plans showed minimal attention to leveraging synergies with existing NCD infrastructure and strategies.

# **Element 8: Proposed financing interventions to enhance CVD control**

With a median of 1.44 [IQR: 1.00, 2.00], financing interventions showed limited comprehensiveness across the 45 countries with significant variation across sub-elements. Allocation of funds and channeling of funds performed relatively better, while resource mobilization showed critical gaps.

## **Cost Measurement Systems sub-element**

38.6% (17/45) of countries scored 1, mentioning the concept of cost but having no system in place to assess the cost of CVD services such as cost per cardiac rehabilitation program or cost per hospital admission for acute MI. 36.4% (16/45) scored 0, providing no discussion. 18.2% (8/45) achieved score 4 with detailed activity-based costing. 6.8% (3/45) scored 2. No countries scored 3 or 5. Median score: 1.0, showing limited systematic cost measurement in CVD planning.

## **Current Financing and Fiscal Space sub-element**

52.3% (23/45) of countries scored 0, indicating no discussion of current CVD financing or fiscal space analysis. 25.0% (11/45) scored 3, providing current spending disaggregated by some recommended categories such as type of CVD or level of facility. 20.5% (9/45) achieved score 4 with comprehensive disaggregation and fiscal space assessment. Only 2.3% (1/45) scored 2. No countries scored 1 or 5. Median score: 0.0, highlighting significant gaps in current financing assessment.

## **Proposed Funding to Implement Plan sub-element**

86.4% (38/45) of countries scored 2, providing a clear funding range to implement the CVD plan. This high concentration demonstrates consistent budgetary planning across most countries. 11.4% (5/45) achieved score 4 with detailed budgets allocating funds to different strategic areas. 2.3% (1/45) scored 3. No countries scored 0, 1, or 5. Median score: 2.0, indicating most plans provide clear funding expectations.

## **Sources of Funds sub-element**

63.6% (28/45) of countries scored 0, indicating no mention of new or expanded funding sources for CVD care such as innovative financing mechanisms. 27.3% (12/45) achieved score 4, quantifying multiple public and private funding sources including domestic and international funding. 9.1% (4/45) scored 3 listing sources without quantification. No countries scored 1, 2, or 5. Median score: 0.0, showing funding source identification remains limited.

## **Pooling of Funds sub-element**

50.0% (22/45) of countries scored 1, mentioning fund pooling but providing no additional details on mechanisms for CVD services. 38.6% (17/45) scored 0, providing no discussion. 4.5% (2/45) scored 2, and 6.8% (3/45) scored 3 with detailed pooling mechanisms described. No countries achieved scores 4 or 5. Median score: 1.0, indicating limited pooling strategy development.

## **Channeling of Funds sub-element**

43.2% (19/45) of countries scored 1, vaguely mentioning entities that will receive CVD funding without specific detail. 40.9% (18/45) achieved score 4, identifying all key entities at different health system levels that will receive funding. 9.1% (4/45) scored 3, 4.5% (2/45) scored 0, and 2.3% (1/45) achieved score 5 with detailed disbursement mechanisms. No countries scored 2. Median score: 3.0, showing moderate specification of funding channels.

## **Allocation of Funds sub-element**

50.0% (22/45) of countries achieved score 4, providing detailed fund allocation to specific strategic priorities with rationale linking to plan objectives. This represents one of the stronger financing sub-elements. 22.7% (10/45) each scored 0 and 3. 4.5% (2/45) scored 1. No countries scored 2 or 5. Median score: 4.0, demonstrating strong allocation planning in many countries.

## **Payment Mechanisms for Providers sub-element**

56.8% (25/45) of countries scored 0, indicating no discussion of how CVD service providers will be remunerated or how payment mechanisms could be reformed to incentivize quality care. 31.8% (14/45) scored 3, discussing how provider payments could change to align with plan goals. 9.1% (4/45) scored 1, and 2.3% (1/45) achieved score 4 with specific payment reform commitments. No countries scored 2 or 5. Median score: 0.0, showing payment mechanism planning remains underdeveloped.

## **Payment Mechanisms for Capital Investments sub-element**

38.6% (17/45) of countries scored 0, indicating no discussion of value-based procurement for CVD capital investments such as diagnostic equipment or facility construction. 9.1% (4/45) scored 3, identifying value-based procurement as an intention. 2.3% (1/45) each scored 1 and 5. Median score: 0.0, highlighting limited value-based investment planning.

## **Resource Mobilization Strategies sub-element**

65.9% (29/45) of countries scored 0, indicating no discussion of resource mobilization strategies for CVD plan implementation such as donor engagement, private sector partnerships, or innovative financing mechanisms. 34.1% (15/45) scored 1, mentioning mobilization strategies without indicators or implementation plans. No countries achieved scores 2 through 5. Median score: 0.0, representing the weakest financing sub-element.

# **Element 9: Proposed resource management interventions to enhance CVD control**

With a median of 1.22 [IQR: 0.67, 1.88], resource management showed limited comprehensiveness across the 45 countries with notable variation. Research and supply chain management performed strongly, while education initiatives and infrastructure showed significant gaps.

## **Human Resources sub-element**

40.9% (18/45) of countries scored 0, indicating no discussion of human resources for CVD control such as workforce needs, training requirements, or task-shifting strategies. 36.4% (16/45) scored 1, mentioning workforce needs without quantification by healthcare worker type. 6.8% (3/45) each scored 2 and 5, the latter providing comprehensive workforce planning with geographic deployment specifications. 2.3% (1/45) scored 4. No countries scored 3. Median score: 1.0, showing human resource planning remains underdeveloped.

## **Infrastructure sub-element**

63.6% (28/45) of countries scored 0, indicating no discussion of infrastructure needs for CVD services such as cardiac catheterization labs, rehabilitation facilities, or diagnostic equipment. 18.2% (8/45) scored 1, 4.5% (2/45) each scored 2 and 4, and 2.3% (1/45) each scored 3 and 5. Median score: 0.0, highlighting significant gaps in infrastructure planning.

## **Information Technology and Data Systems sub-element**

15.9% (7/45) of countries scored 0, indicating no discussion of IT and data systems for CVD care coordination such as electronic health records, telemedicine platforms, or health information exchanges. 13.6% (6/45) scored 1. 4.5% (2/45) scored 2. 9.1% (4/45) scored 4, and 4.5% (2/45) achieved score 5 with comprehensive digital health infrastructure plans including interoperability standards. Median score: 1.0, showing variable attention to digital health infrastructure.

## **Pharmaceuticals and Medical Supplies sub-element**

61.4% (27/45) of countries scored 0, indicating no discussion of pharmaceutical needs or targets for essential CVD medications such as antihypertensives, statins, aspirin, and antiplatelets on the essential medicines list. 15.9% (7/45) achieved score 5 with comprehensive supply security strategies addressing local production and quality assurance. 13.6% (6/45) scored 1, 6.8% (3/45) scored 2, and 2.3% (1/45) scored 4. No countries scored 3. Median score: 0.0, showing pharmaceutical access planning remains a significant gap.

## **Supply Chain Management sub-element**

22.7% (10/45) of countries scored 4, indicating comprehensive supply chain planning addressing most critical aspects of procurement, distribution, storage, and inventory management for CVD commodities. 13.6% (6/45) each scored 0 and 5, the latter addressing all aspects with performance monitoring. 2.3% (1/45) scored 1. No countries scored 2 or 3. Median score: 4.0, representing one of the stronger resource management sub-elements.

## **Research sub-element**

68.2% (30/45) of countries achieved score 4, indicating comprehensive research initiative planning with priority areas, capacity-building needs, and implementation targets for CVD research including clinical trials, health services research, and implementation science. This represents the strongest resource management sub-element. 15.9% (7/45) scored 0, 6.8% (3/45) scored 3, and 4.5% (2/45) each scored 1 and 5. No countries scored 2. Median score: 4.0.

## **Innovation Ecosystem sub-element**

43.2% (19/45) of countries each scored 0 and 1, indicating either no discussion or basic mention of innovation ecosystem development for CVD such as partnerships with technology companies, incubators for digital health solutions, or incentives for local innovation. 2.3% (1/45) each scored 2, 3, 4, and 5. Median score: 1.0, showing limited systematic planning for CVD innovation.

## **Capacity Building for Health Professionals sub-element**

56.8% (25/45) of countries scored 0, indicating no discussion of capacity building initiatives for health professionals in CVD prevention, diagnosis, treatment, and management such as continuing medical education, specialized training programs, or competency frameworks. 22.7% (10/45) scored 3 with comprehensive training programs described. 11.4% (5/45) scored 2, and 9.1% (4/45) scored 1. No countries achieved scores 4 or 5. Median score: 0.0, highlighting a critical gap in workforce development.

## **Education Initiatives sub-element**

75.0% (33/45) of countries scored 0, indicating no discussion of educational initiatives for CVD awareness, prevention, or health literacy at the population level such as public awareness campaigns, school-based education, or community health education programs. 15.9% (7/45) scored 1, and 6.8% (3/45) scored 2. No countries achieved scores 3, 4, or 5. Median score: 0.0, representing one of the weakest sub-elements across all CVD plans.

# **Element 10: Proposed changes in health services delivery to enhance CVD control**

With a median of 1.43 [IQR: 1.00, 1.86], health services delivery showed moderate comprehensiveness across the 45 countries with significant variation. Diagnostic services and provider value enhancement performed relatively well, while health promotion and protection showed substantial gaps.

## **Personal Healthcare Services (Disease Prevention) sub-element**

61.4% (27/45) of countries scored 0, indicating no discussion of screening services for CVD risk factors such as systematic hypertension screening, diabetes screening, or dyslipidemia assessment at the primary care level. 13.6% (6/45) achieved score 4 with comprehensive screening programs including target populations, frequency, and follow-up protocols. 9.1% (4/45) scored 2, 6.8% (3/45) each scored 1 and 3, and 2.3% (1/45) achieved score 5. Median score: 0.0, highlighting significant gaps in secondary prevention planning.

## **Public Health Services (Health Promotion) sub-element**

56.8% (25/45) of countries scored 0, indicating no discussion of health promotion interventions for CVD risk factors such as tobacco cessation programs, healthy diet promotion, sodium reduction initiatives, or physical activity campaigns. 13.6% (6/45) each scored 2 and 3. 9.1% (4/45) achieved score 5 with comprehensive strategies addressing all major risk factors with evaluation frameworks. 6.8% (3/45) scored 4. No countries scored 1. Median score: 0.0, showing primary prevention through health promotion remains underdeveloped.

## **Personal Healthcare Services (Diagnosis) sub-element**

54.5% (24/45) of countries scored 2, indicating discussion of evidence-based diagnostics for some major CVD conditions with relevant indicators such as availability of ECG, echocardiography, or cardiac biomarker testing. This represents one of the stronger health services sub-elements. 18.2% (8/45) scored 1, 9.1% (4/45) each scored 0 and 3, and 4.5% (2/45) achieved score 4 with comprehensive diagnostic service planning. No countries achieved score 5. Median score: 2.0.

## **Personal Healthcare Services (Treatment) sub-element**

31.8% (14/45) of countries scored 0, indicating no discussion of treatment services for CVD such as acute coronary syndrome management, heart failure treatment, or stroke care pathways. 25.0% (11/45) scored 2 with some evidence-based treatments described. 22.7% (10/45) scored 3, 15.9% (7/45) scored 4 with comprehensive treatment planning, and 2.3% (1/45) each scored 1 and 5. Median score: 2.0, showing moderate but variable treatment service planning.

## **Personal Healthcare Services (Palliation and Rehabilitative Care) sub-element**

59.1% (26/45) of countries scored 0, indicating no discussion of cardiac rehabilitation or palliative care services for advanced CVD such as phase I-III rehabilitation programs, exercise-based cardiac rehabilitation, or end-of-life care pathways. 29.5% (13/45) scored 3 with comprehensive rehabilitation programs described including patient selection criteria and outcome measurement. 6.8% (3/45) achieved score 4 with detailed implementation plans. No countries scored 1, 2, or 5. Median score: 0.0, highlighting a significant gap in rehabilitative and palliative care planning.

## **Public Health Services (Health Protection) sub-element**

81.8% (36/45) of countries scored 0, indicating no discussion of health protection interventions for CVD such as tobacco taxation and smoke-free policies, regulation of trans fats and sodium in processed foods, restrictions on marketing of unhealthy foods to children, or air quality improvements. This high proportion at score 0 represents the weakest health services sub-element. 9.1% (4/45) scored 3, and 4.5% (2/45) each scored 1 and 2. No countries achieved scores 4 or 5. Median score: 0.0.

## **Provider Value Enhancement sub-element**

27.3% (12/45) of countries achieved score 4, indicating plans to implement most distinct value-enhancement measures such as electronic health records, cost measurement systems, clinical pathways for CVD conditions, and performance benchmarking. 25.0% (11/45) scored 3 with several measures described. 18.2% (8/45) scored 0, 13.6% (6/45) each scored 1 and 2, and 2.3% (1/45) achieved score 5 with comprehensive value-based care transformation. Median score: 3.0, showing relatively stronger performance in value enhancement planning.

# **Element 11: Implementation of the National CVD Control Plan**

With a median of 1.75 [IQR: 0.50, 2.50], implementation planning showed moderate comprehensiveness across the 45 countries with significant variation across domains. Risk and mitigation showed a notably bimodal distribution.

## **Stakeholder Engagement sub-element**

47.7% (21/45) of countries scored 0, indicating no discussion of stakeholder engagement strategies for CVD plan implementation such as identification of key stakeholders, engagement mechanisms, or communication strategies. 18.2% (8/45) each scored 2 and 4. 9.1% (4/45) scored 1, 4.5% (2/45) scored 3, and 2.3% (1/45) achieved score 5 with comprehensive stakeholder engagement strategy linked to governance structures and accountability mechanisms. Median score: 1.0, showing stakeholder engagement remains underdeveloped in most plans.

## **Monitoring and Evaluation Framework sub-element**

43.2% (19/45) of countries scored 0, indicating no monitoring and evaluation plan for CVD control with indicators, timelines, or responsibilities. 34.1% (15/45) scored 2, providing M&E plans missing some critical aspects such as baseline data or evaluation methodology. 11.4% (5/45) achieved score 4 with complete M&E frameworks including activities, outputs, outcomes, indicators, timelines, and responsibilities. 9.1% (4/45) scored 1, and 2.3% (1/45) achieved score 5 with linkage to continuous quality improvement. No countries scored 3. Median score: 1.0, showing M&E framework development remains inconsistent.

## **Risk and Mitigation Strategies sub-element**

The distribution was notably bimodal: 59.1% (26/45) of countries scored 0, indicating no identification or analysis of risks to CVD plan implementation such as political changes, funding shortfalls, stakeholder resistance, or health system capacity constraints. However, 34.1% (15/45) achieved score 5, providing detailed risk registers including risk probability and impact analysis, specific mitigation measures for each risk, and assigned monitoring responsibilities with escalation procedures. Only 6.8% (3/45) scored 1. No countries scored 2, 3, or 4. Median score: 0.0, showing most plans either omit risk analysis entirely or provide comprehensive frameworks.

## **Change Management sub-element**

36.4% (16/45) of countries scored 1, mentioning change management approaches but not describing critical aspects such as stakeholder readiness assessment, leadership engagement strategy, communication plans, or resistance management. 25.0% (11/45) achieved score 4 with comprehensive approaches covering all critical aspects including staff training and organizational culture considerations. 15.9% (7/45) scored 0, 6.8% (3/45) scored 2, and 2.3% (1/45) scored 3. No countries achieved score 5 with detailed implementation timelines and success metrics. Median score: 1.0, indicating change management processes require significant strengthening across CVD plans.
