# Supplementary material for: A global analysis of national cardiovascular disease control plans using a multi-agent artificial intelligence model
Source: PLOS Digit Health. 2026 Jun 1;5(6):e0001447. doi: 10.1371/journal.pdig.0001447 (PMC13225395; doi:10.1371/journal.pdig.0001447)
Supplement: S1 Table — (DOCX) [file pdig.0001447.s006.docx]

**S1: National Cardiovascular Disease Control Plan Scoring Rubric**

| **ELEMENT 1: HEALTH SYSTEM PERFORMANCE OUTCOMES** | **Description** |  |  |  | **Score** |  |  | **Example Indicators** |
| --- | --- | --- | --- | --- | --- | --- | --- | --- |
| **How well is the health system performing in relation to care and control concerning health system outcomes for cardiovascular disease?** |  | **0** | **1** | **2** | **3** | **4** | **5** |  |
| **Health** | How the health system is currently performing in relation to cardiovascular disease (CVD), assessed in terms of health outcomes. These include traditional epidemiological metrics as well as patient-reported health status, quality of life, and overall wellbeing. This assessment should also account for the impact of key comorbidities and multimorbidity, including explicit consideration of pregnancy-related cardiovascular complications. | Plan does not discuss any health outcomes and does not use indicators to measure or assess. | Plan discusses some health outcomes but does not use specific indicators to assess them. | Plan discusses some health outcomes and uses appropriate indicators to assess them. | Plan discusses all health outcomes and uses appropriate indicators comprehensively to assess them. | Plan discusses all health outcomes, uses appropriate indicators comprehensively, and provides national baseline data for these outcomes. | Plan discusses all health outcomes, uses appropriate indicators comprehensively with baseline data, and includes specific, measurable targets for performance improvement. | - CVD-specific mortality rate (age-standardized), disaggregated by geography (e.g., urban/rural), socioeconomic status, and life-course stage - Prevalence of CVD (age-standardized), similarly disaggregated - Incidence of key CVD events (e.g., MI, stroke) per 100,000 - Years free of major adverse cardiovascular events - Years with controlled cardiovascular risk factors (e.g., BP, LDL, HbA1c within target) - 5-year survival rate for acute MI - DALYs lost due to CVD - QALYs gained from CVD interventions - Patient-reported outcomes for health status and wellbeing (e.g., KCCQ for heart failure) - Prevalence and treatment rates of key associated conditions (e.g., periodontal disease) within the CVD population - Proportion of total mortality due to cardiovascular disease - Screening rates for cardiovascular complications during pregnancy and postpartum period |
| **Financial Risk Protection** | How the health system is currently performing in relation to CVD, assessed in terms of financial risk protection outcomes, which include: levels of out-of-pocket health expenditures as a percentage of total health spending, levels catastrophic or impoverishing health expenditures, levels of impoverishing health expenditures by income quintiles. | Plan does not discuss any outcomes related to financial risk protection and does not use indicators to measure or assess. | Plan discusses some outcomes related to financial risk protection but does not use specific indicators. | Plan discusses some outcomes related to financial risk protection and uses appropriate indicators. | Plan discusses all outcomes related to financial risk protection and uses appropriate indicators comprehensively. | Plan discusses all outcomes related to financial risk protection, uses appropriate indicators comprehensively, and provides national baseline data. | Plan discusses all outcomes related to financial risk protection, uses appropriate indicators comprehensively with baseline data and includes specific targets for reducing financial burden. | - Out-of-pocket payments as % of Total Health Expenditure for CVD services - Incidence of Catastrophic Health Expenditure for CVD care, Incidence of Impoverishing Health Expenditure from CVD care - Life years saved from CVD interventions - Productivity gains from CVD treatment (working years gained) - Healthcare utilization metrics |
| **User Satisfaction** | How the health system is currently performing in relation to CVD, assessed in terms of user satisfaction, which include: autonomy, choice, communication, confidentiality, dignity, prompt attention, trust, quality of basic amenities and support. | Plan does not discuss any outcomes related to user satisfaction and does not use indicators to measure or assess. | Plan discusses some outcomes related to user satisfaction but does not use specific indicators. | Plan discusses some outcomes related to user satisfaction and uses appropriate indicators. | Plan discusses all outcomes related to user satisfaction and uses appropriate indicators comprehensively. | Plan discusses all outcomes related to user satisfaction, uses appropriate indicators comprehensively, and provides national baseline data from patient surveys. | Plan discusses all outcomes related to user satisfaction, uses appropriate indicators comprehensively with baseline data, and includes specific targets for improving the patient experience. | - Patient-reported experience measures for CVD care - Measures of shared decision-making in treatment choices (e.g., choice of cardiac rehab model) - Patient trust in health providers and the health system. |
| **ELEMENT 2: HEALTH SYSTEM PERFORMANCE OBJECTIVES** | **Description** |  |  |  | **Score** |  |  | **Example Indicators** |
| **How well is the health system producing health services for CVD in terms of the four value dimensions?** |  | **0** | **1** | **2** | **3** | **4** | **5** |  |
| **Effectiveness** | How the health system is currently performing in relation to CVD, assessed in terms of the delivery of health services that are effective at improving health. This includes controlling key metabolic risk factors, implementing evidence-based treatments, and integrating the management of key comorbidities that increase cardiovascular risk. | Plan does not discuss the effectiveness of health services for CVD and does not use indicators to measure or assess. | Plan discusses health service effectiveness in relation to CVD but does not use specific indicators. | Plan discusses health service effectiveness in relation to CVD and uses appropriate indicators. | Plan discusses health service effectiveness in relation to CVD and uses appropriate indicators comprehensively. | Plan discusses health service effectiveness, uses appropriate indicators comprehensively to assess performance, and provides national baseline data for these indicators. | Plan discusses health service effectiveness, uses appropriate indicators comprehensively with baseline data, includes specific targets for improvement, and outlines quality improvement strategies. | - Percentage of hypertensive patients with controlled blood pressure (<140/90 mmHg). - Percentage of high-risk patients with controlled LDL-cholesterol according to guideline-recommended targets. - Proportion of eligible patients prescribed appropriate secondary prevention medications (e.g., antiplatelets, statins, ACE-inhibitors, beta-blockers). - Uptake and completion rates for cardiac rehabilitation among eligible patients. - Influenza vaccination rate among patients with established CVD. |
| **Efficiency** | How the health system is currently performing in relation to CVD, assessed in terms of the delivery of health services that are efficient, maximizing health outcomes relative to the resources used. | Plan does not discuss the efficiency of health services for CVD and does not include indicators to measure performance. | Plan discusses health service efficiency in relation to CVD but without specific indicators. | Plan discusses health service efficiency in relation to CVD and includes appropriate indicators. | Plan discusses health service efficiency in relation to CVD and includes appropriate indicators comprehensively. | Plan discusses health service efficiency, includes appropriate indicators comprehensively to assess performance, and provides national baseline data where applicable. | Plan discusses health service efficiency, includes appropriate indicators comprehensively with baseline data, and includes specific targets for improving the use of resources. | - Average length of stay for CVD-related hospitalizations - Cost per CVD-related hospitalization - Proportion of generic vs. brand-name CVD drugs prescribed - Bed occupancy rate in cardiac care units - Rate of hospitalizations for ambulatory-care-sensitive conditions - Metric of resource use vs. reduction in severe cardiovascular events - Cost-effectiveness of preventive oral interventions in reducing CVD complications |
| **Equity** | How the health system is currently performing in relation to CVD, assessed in terms of the delivery of health services that are equitable, ensuring that access, quality of care, and outcomes do not vary across population subgroups. This includes explicit consideration of gender disparities in CVD prevention, diagnosis, treatment, and outcomes, recognizing that cardiovascular disease presents differently and is often under-diagnosed in women. | Plan does not discuss the equity of health services for CVD and does not use indicators to measure or assess. | Plan discusses health service equity in relation to CVD but does not use specific indicators or disaggregated data. | Plan discusses health service equity in relation to CVD and uses appropriate indicators to highlight disparities. | Plan discusses health service equity in relation to CVD and uses appropriate indicators comprehensively to highlight disparities. | Plan discusses health service equity, uses appropriate indicators comprehensively, and provides disaggregated national baseline data demonstrating existing disparities. | Plan discusses health service equity, uses disaggregated baseline data, and includes specific targets and tailored interventions aimed at reducing identified health inequities. | - Disparities in CVD mortality rates by socioeconomic status - Disparities in access to cardiac rehabilitation by geographic location - Differences in control rates (BP, cholesterol) by ethnicity - Wait times for cardiac procedures disaggregated by insurance type - Gap in hypertension/diabetes/LDL control across socioeconomic & geographic groups - Percentage of patients in rural/vulnerable areas with access to essential CVD medications - Number of municipalities meeting minimum primary care coverage - Gender gap in time from symptom onset to treatment for acute MI - Gender differences in prescription rates of evidence-based secondary prevention medications - Gender differences in referral rates to cardiac rehabilitation |
| **Responsiveness** | How the health system is currently performing in relation to CVD, assessed in terms of its ability to meet the legitimate non-health expectations of users. This includes respect for persons, client orientation, timeliness of care, and patient-centred communication. | Plan does not discuss the responsiveness of health services for CVD and does not include indicators to measure performance. | Plan discusses health service responsiveness in relation to CVD but without specific indicators. | Plan discusses health service responsiveness in relation to CVD and includes appropriate indicators. | Plan discusses health service responsiveness in relation to CVD and includes appropriate indicators comprehensively. | Plan discusses health service responsiveness, includes appropriate indicators comprehensively to assess performance, and provides national baseline data for these indicators. | Plan discusses health service responsiveness, includes appropriate indicators comprehensively with baseline data, and includes specific targets for improving system responsiveness for patients with CVD. | - Patient satisfaction scores with CVD care - Average wait time for cardiology appointments - Percentage of patients reporting good communication with their provider - Number of patient complaints related to cardiac care - Percentage of patients reporting clear communication and understanding of treatment - Percentage of health facilities with certified humanized care |
| **ELEMENT 3: HEALTH SYSTEM PERFORMANCE OUTPUTS** | **Description** |  |  |  | **Score** |  |  | **Example Indicators** |
| **Is the health system delivering the right health services to respond to cardiovascular disease?** |  | **0** | **1** | **2** | **3** | **4** | **5** |  |
| **Individual Health Services** | Describes the performance of health system outputs, assessed in terms of the delivery, integration, and care model of individual health services for CVD provided to patients at primary, secondary, and tertiary levels. Plans should specify the level of care at which services are delivered and identify which personnel are responsible for managing different conditions at each level, particularly for common conditions like hypertension. | Plan does not discuss individual health services for CVD and does not use indicators to measure or assess. | Plan discusses some individual health services for CVD but does not use specific indicators to measure output. | Plan discusses some individual health services for CVD and uses appropriate indicators to measure output. | Plan discusses all key individual health services for CVD and uses appropriate indicators comprehensively to measure output. | Plan discusses all key individual health services, uses appropriate indicators comprehensively, and provides national baseline data on the volume of these services. | Plan discusses all key individual health services, uses appropriate indicators comprehensively with baseline data, and includes specific, measurable targets for service delivery. | - Availability of essential CVD diagnostics (e.g., ECG, troponin) at primary care level - Number of cardiac catheterization labs per million population - Proportion of primary care facilities offering CVD risk assessment - Availability of cardiac rehabilitation programs - Availability and integration of multidisciplinary care teams (including dental, nursing, etc.) - Proportion of CVD care pathways that integrate oral health assessments - Percentage of hypertension cases managed at primary care level (without specialist referral) - Availability of virtual/remote cardiac rehabilitation programs - Adoption rate of digital health technologies for CVD monitoring and management |
| **Population Health Services** | Describes the performance of health system outputs, assessed in terms of the delivery of population-level health services for CVD prevention and control. Plans should specify the location and model of service delivery to address capacity constraints in overburdened health systems. | Plan does not discuss population health services for CVD and does not use indicators to measure or assess. | Plan discusses some population health services for CVD but does not use specific indicators to measure output. | Plan discusses some population health services for CVD and uses appropriate indicators to measure output. | Plan discusses all key population health services for CVD and uses appropriate indicators comprehensively to measure output. | Plan discusses all key population health services, uses appropriate indicators comprehensively, and provides national baseline data on the reach and availability of these services. | Plan discusses all key population health services, uses appropriate indicators comprehensively with baseline data, and includes specific, measurable targets for expanding service coverage. | - Coverage rate of national smoking cessation programs  Proportion of schools with healthy food policies  Availability of public spaces for physical activity  Coverage of salt reduction initiatives  Existence and coverage of dedicated community-level CVD/NCD screening programs - Percentage of patients with obesity who receive specific obesity treatment - Proportion of prevention services delivered in community settings vs. clinic settings - Availability of transportation support programs or regulations facilitating access to CVD services |
| **Community-Based Palliative Care** | Assesses the delivery and integration of community-based palliative and supportive care services for patients with advanced CVD. This focuses on improving quality of life for patients and their families through the management of symptoms like dyspnea, pain, and fatigue, as well as providing psychosocial, spiritual, and care-planning support in home or community settings. | Plan does not discuss any community-based palliative care services for CVD and does not include indicators to measure performance to assess these services | Plan discusses some community-based palliative care services for CVD but without specific indicators to assess these services | Plan discusses some community-based palliative care services for CVD and includes appropriate indicators to assess these services | Plan discusses all community-based palliative care services for CVD and includes appropriate indicators comprehensively to assess these services | Plan discusses all community-based palliative care services for CVD, includes appropriate indicators comprehensively to assess these services, and includes targets demonstrating performance | Plan discusses all community-based palliative care services for CVD, includes appropriate indicators comprehensively to assess these services, includes targets demonstrating performance, and links how health system outputs, objectives, and inputs have contributed to these outcomes | - Percentage of CVD patients receiving home-based palliative care - Availability of oral morphine in community health settings - Number of trained community health workers providing palliative care per 100,000 population - Percentage of palliative care patients with documented pain relief |
| **CVD Surveillance Systems** | How the health system is currently performing in relation to CVD, assessed in terms of the establishment and operation of CVD surveillance systems, including population-based registries for key CVD events and risk factor monitoring, to measure the burden of CVD and inform policy and planning. | Plan does not discuss any CVD surveillance systems and does not include indicators to measure performance to assess surveillance outcomes | Plan discusses some CVD surveillance systems but without specific indicators to assess surveillance outcomes | Plan discusses some CVD surveillance systems and includes appropriate indicators to assess surveillance outcomes | Plan discusses all CVD surveillance systems, including population-based CVD registries and risk factor monitoring, and includes appropriate indicators comprehensively to assess surveillance outcomes | Plan discusses all CVD surveillance systems, includes appropriate indicators comprehensively to assess surveillance outcomes, and includes targets demonstrating performance | Plan discusses all CVD surveillance systems, includes appropriate indicators comprehensively to assess surveillance outcomes, includes targets demonstrating performance, and links how surveillance outputs, objectives, and inputs have contributed to these outcomes | - Existence of a national population-based CVD registry (e.g., for MI, stroke) - Existence of an out-of-hospital cardiac arrest registry - Completeness and quality of vital registration data for CVD mortality - Frequency of national risk factor surveys (e.g., STEPS) |
| **ELEMENT 4: HEALTH SYSTEM THREATS** | **Description** |  |  |  | **Score** |  |  | **Example Indicators** |
| **What contextual threats will influence CVD demands on the health system and influence the system response to CVD care and control?** |  | **0** | **1** | **2** | **3** | **4** | **5** |  |
| **Demographic** | Demographic factors that pose a threat to CVD control, such as an aging population or a large youth population adopting unhealthy lifestyles, which increases the overall prevalence and complexity of cardiovascular diseases. | Plan does not discuss any demographic threats. | Plan discusses some demographic threats but does not include indicators to measure performance. | Plan discusses some demographic threats and includes appropriate indicators. | Plan discusses all relevant demographic threats and includes appropriate indicators. | Plan discusses all relevant demographic threats and includes appropriate indicators comprehensively to quantify them. | Plan discusses all demographic threats, uses all indicators, and provides an analysis of the certainty, magnitude, and timing of their impact on the health system. | - Population growth rate - Percentage of population over 65 years - Dependency ratio - Urban vs. rural population distribution |
| **Epidemiologic** | Epidemiological factors that pose a threat to CVD control, including the high or rising prevalence of CVD and its underlying metabolic, behavioural, and environmental risk factors in the population. Epidemiological factors that pose a threat to CVD control, including the high or rising prevalence of CVD and its underlying metabolic, behavioural, and environmental risk factors in the population. | Plan does not discuss any epidemiologic threats. | Plan discusses some epidemiologic threats but does not include indicators to measure performance. | Plan discusses some epidemiologic threats and includes appropriate indicators. | Plan discusses all relevant epidemiologic threats and includes appropriate indicators. | Plan discusses all relevant epidemiologic threats and includes appropriate indicators comprehensively to quantify them. | Plan discusses all epidemiologic threats, uses all indicators, and provides an analysis of the certainty, magnitude, and timing of their impact on the health system. | - Prevalence of hypertension - Prevalence of diabetes - Prevalence of obesity/overweight - Prevalence of dyslipidaemia - Prevalence of smoking - Prevalence of physical inactivity - Prevalence of unhealthy diet (e.g., high sodium intake) |
| **Political** | Political factors that pose a threat to CVD control, such as government instability, a lack of political commitment to NCDs, or shifting priorities and funding away from primary healthcare and prevention. | Plan does not discuss any political threats. | Plan discusses some political threats but does not include indicators to measure performance. | Plan discusses some political threats and includes appropriate indicators. | Plan discusses all relevant political threats and includes appropriate indicators. | Plan discusses all relevant political threats and includes appropriate indicators comprehensively to assess them. | Plan discusses all political threats, uses all indicators, and provides an analysis of the certainty, magnitude, and timing of their impact on the health system. | - Degree of political stability and governance effectiveness. - Lack of a national NCD/CVD action plan or strategy. |
| **Legal** | The absence of a supportive legal and regulatory framework that could mitigate CVD risk factors and strengthen the health system's response. | Plan does not discuss any legal threats. | Plan discusses some legal threats but does not include indicators to measure performance. | Plan discusses some legal threats and includes appropriate indicators. | Plan discusses all relevant legal threats and includes appropriate indicators. | Plan discusses all relevant legal threats and includes appropriate indicators comprehensively to assess them. | Plan discusses all legal threats, uses all indicators, and provides an analysis of the certainty, magnitude, and timing of their impact on the health system. | - Absence of a statutory right to health in the constitution. - Absence of regulations on food labelling (e.g., for sodium, trans-fats, and sugar). - Absence of regulations restricting the advertising of unhealthy foods and beverages to children. - Weak or poorly enforced tobacco control legislation. - Lack of regulation for e-cigarettes. |
| **Sociocultural** | Sociocultural factors that pose a threat to CVD control, including societal norms, beliefs, and practices that promote unhealthy behaviours, as well as structural biases that create health disparities. | Plan does not discuss any sociocultural threats. | Plan discusses some sociocultural threats but does not include indicators to measure performance. | Plan discusses some sociocultural threats and includes appropriate indicators. | Plan discusses all relevant sociocultural threats and includes appropriate indicators. | Plan discusses all relevant sociocultural threats and includes appropriate indicators comprehensively to assess them. | Plan discusses all sociocultural threats, uses all indicators, and provides an analysis of the certainty, magnitude, and timing of their impact on the health system. | - Population health literacy levels. - Prevalence of tobacco use (% of adults). - Per capita alcohol consumption. - Evidence of structural bias (racism, gender bias) leading to health disparities in CVD care. - Prevalence of health-related misinformation and lack of public confidence in preventative medicine. - Population-level scores for CVD risk awareness |
| **Economic** | Economic factors that pose a threat to CVD control, such as economic instability, poverty, disruptive global trade/tariffs, and the high cost of care, often exacerbated by a lack of domestic manufacturing capacity for essential medicines and technologies. | Plan does not discuss any economic threats. | Plan discusses some economic threats but does not include indicators to measure performance. | Plan discusses some economic threats and includes appropriate indicators. | Plan discusses all relevant economic threats and includes appropriate indicators. | Plan discusses all relevant economic threats and includes appropriate indicators comprehensively to assess them. | Plan discusses all economic threats, uses all indicators, and provides an analysis of the certainty, magnitude, and timing of their impact on the health system. | - GDP growth rate. - GINI co-efficient (income inequality). - National poverty level. - Low availability and affordability of essential secondary prevention medicines (including generics and polypills). - High patient out-of-pocket costs or co-payments for CVD medicines and care. - Reliance on importation for essential CVD medicines - Impact of global trade tariffs on medicine costs |
| **Ecological** | Ecological and environmental factors that pose a threat to CVD control, including air pollution, noise pollution, light pollution, and urban environments that are not supportive of healthy lifestyles. | Plan does not discuss any ecological threats. | Plan discusses some ecological threats but does not include indicators to measure performance. | Plan discusses some ecological threats and includes appropriate indicators. | Plan discusses all relevant ecological threats and includes appropriate indicators. | Plan discusses all relevant ecological threats and includes appropriate indicators comprehensively to assess them. | Plan discusses all ecological threats, uses all indicators, and provides an analysis of the certainty, magnitude, and timing of their impact on the health system. | - Urban air quality index (e.g., average PM2.5 levels). - Impact of climate change and extreme weather events (e.g., heatwaves) on vulnerable populations and health services. - Poor urban planning leading to low 'walkability' and limited access to green spaces for physical activity. - High density of fast-food outlets, particularly in low-income areas. - Population exposure to high decibel levels (noise pollution) |
| **Technological** | Technological factors that pose a threat to CVD control, such as the "digital divide" limiting access to digital health tools, a lack of system interoperability, and the potential for technology to spread health misinformation. | Plan does not discuss any technological threats. | Plan discusses some technological threats but does not include indicators to measure performance. | Plan discusses some technological threats and includes appropriate indicators. | Plan discusses all relevant technological threats and includes appropriate indicators. | Plan discusses all relevant technological threats and includes appropriate indicators comprehensively to assess them. | Plan discusses all technological threats, uses all indicators, and provides an analysis of the certainty, magnitude, and timing of their impact on the health system. | - Low or inequitable internet and smartphone penetration rates. - Lack of interoperability between different electronic health records (e.g., between primary and secondary care). - Evidence of widespread health misinformation related to CVD prevention (e.g., regarding vaccines or statins). - Potential for safety concerns or bias in conversational AI and other emerging technologies. |
| **ELEMENT 5: HEALTH SYSTEM OPPORTUNITIES** | **Description** |  |  |  | **Score** |  |  | **Example Indicators** |
| **What contextual opportunities will influence CVD demands on the health system and influence the system response to CVD care and control?** |  | **0** | **1** | **2** | **3** | **4** | **5** |  |
| **Demographic** | Demographic factors that present an opportunity for CVD control, such as a large youth population allowing for a focus on primary prevention before CVD becomes highly prevalent. | Plan does not discuss any demographic opportunities. | Plan discusses some demographic opportunities but does not include indicators to measure performance. | Plan discusses some demographic opportunities and includes appropriate indicators. | Plan discusses all relevant demographic opportunities and includes appropriate indicators. | Plan discusses all relevant demographic opportunities and includes appropriate indicators comprehensively to quantify them. | Plan discusses all relevant demographic opportunities and includes appropriate indicators comprehensively to quantify them. | - Favourable dependency ratio (large working-age population). - High proportion of population under 15 years, allowing for early intervention. - Increasing urbanization, which can be leveraged to centralize specialized services. |
| **Epidemiologic** | Epidemiological factors that present an opportunity for CVD control, such as low or stabilizing prevalence of key risk factors, or high population awareness of CVD that can be leveraged for health campaigns. This also includes the opportunity to integrate prevention and management of key modifiable risk factors into routine CVD care. | Plan does not discuss any epidemiologic opportunities. | Plan discusses some epidemiologic opportunities but does not include indicators to measure performance. | Plan discusses some epidemiologic opportunities and includes appropriate indicators. | Plan discusses all relevant epidemiologic opportunities and includes appropriate indicators. | Plan discusses all relevant epidemiologic opportunities and includes appropriate indicators comprehensively to quantify them. | Plan discusses all epidemiologic opportunities, uses all indicators, and provides an analysis of the certainty, magnitude, and timing of their potential positive impact. | - Low or stabilizing prevalence of hypertension, diabetes, and obesity. - High population awareness of CVD risk factors. - High rates of influenza vaccination, which can be leveraged for opportunistic CVD prevention messaging. - Existence of programs for integrated oral-cardiovascular prevention |
| **Political** | Political factors that present an opportunity for CVD control, such as strong government stability, high political will to address NCDs, alignment with key opinion leaders, and a commitment to investing in primary healthcare and prevention. | Plan does not discuss any political opportunities. | Plan discusses some political opportunities but does not include indicators to measure performance. | Plan discusses some political opportunities and includes appropriate indicators. | Plan discusses all relevant political opportunities and includes appropriate indicators. | Plan discusses all relevant political opportunities and includes appropriate indicators comprehensively to assess them. | Plan discusses all political opportunities, uses all indicators, and provides an analysis of the certainty, magnitude, and timing of their potential positive impact. | - High degree of political stability and governance effectiveness. - Existence of a well-funded national NCD/CVD action plan or strategy. - Strong and sustained political commitment to investment in primary healthcare. - Existence of a robust, complementary public health infrastructure |
| **Legal** | The existence of a strong legal and regulatory framework that enables public health action, promotes healthy environments, and ensures access to care. | Plan does not discuss any legal opportunities. | Plan discusses some legal opportunities but does not include indicators to measure performance. | Plan discusses some legal opportunities and includes appropriate indicators. | Plan discusses all relevant legal opportunities and includes appropriate indicators. | Plan discusses all relevant legal opportunities and includes appropriate indicators comprehensively to assess them. | Plan discusses all legal opportunities, uses all indicators, and provides an analysis of the certainty, magnitude, and timing of their potential positive impact. | - Existence of a statutory right to health in the constitution. - Comprehensive laws on food labelling, advertising restrictions, and health taxes (e.g., on sugar-sweetened beverages). - Strong and well-enforced tobacco and e-cigarette control legislation. - Inclusion of comprehensive secondary prevention medicines (including fixed-dose combinations/polypill) on the national essential medicines list. |
| **Sociocultural** | Sociocultural factors that present an opportunity for CVD control, such as high health literacy, societal norms that favour healthy lifestyles, and strong community and family and active civil society/patient advocacy group support structures. | Plan does not discuss any sociocultural opportunities. | Plan discusses some sociocultural opportunities but does not include indicators to measure performance. | Plan discusses some sociocultural opportunities and includes appropriate indicators. | Plan discusses all relevant sociocultural opportunities and includes appropriate indicators. | Plan discusses all relevant sociocultural opportunities and includes appropriate indicators comprehensively to assess them. | Plan discusses all sociocultural opportunities, uses all indicators, and provides an analysis of the certainty, magnitude, and timing of their potential positive impact. | - High and equitable health literacy levels in the population. - Low prevalence of tobacco use and harmful alcohol consumption. - Strong social cohesion and community engagement in health programs. - Presence of strong family and caregiver support systems for patients. |
| **Economic** | Economic factors that present an opportunity for CVD control, such as strong economic growth providing fiscal space for health, local manufacturing capacity for affordable medicines and technologies, and the availability of low-cost, highly cost-effective preventative interventions. | Plan does not discuss any economic opportunities. | Plan discusses some economic opportunities but does not include indicators to measure performance. | Plan discusses some economic opportunities and includes appropriate indicators. | Plan discusses all relevant economic opportunities and includes appropriate indicators. | Plan discusses all relevant economic opportunities and includes appropriate indicators comprehensively to assess them. | Plan discusses all economic opportunities, uses all indicators, and provides an analysis of the certainty, magnitude, and timing of their potential positive impact. Existence of domestic manufacturing capacity for essential CVD medicines | - Strong and stable GDP growth. - Low national poverty level and low income-inequality (GINI co-efficient). - Widespread availability and affordability of generic secondary prevention medicines. - Policies that reduce or eliminate co-payments for essential CVD medicines and services. - Established use of health taxes (e.g., tobacco, alcohol, sugar) to generate revenue for health programs. |
| **Ecological** | Ecological and environmental factors that present an opportunity for CVD control, such as urban planning that creates 'healthy settings' supportive of physical activity and healthy diets. | Plan does not discuss any ecological opportunities. | Plan discusses some ecological opportunities but does not include indicators to measure performance. | Plan discusses some ecological opportunities and includes appropriate indicators. | Plan discusses all relevant ecological opportunities and includes appropriate indicators. | Plan discusses all relevant ecological opportunities and includes appropriate indicators comprehensively to assess them. | Plan discusses all ecological opportunities, uses all indicators, and provides an analysis of the certainty, magnitude, and timing of their potential positive impact. | - Good urban air quality. - High 'walkability' scores of cities and towns. - Widespread, safe, and equitable access to green spaces and recreational facilities. - Zoning policies that promote access to healthy food retailers. |
| **Technological** | Technological factors that present an opportunity for CVD control, including the growing availability of digital health tools, widespread mobile/digital coverage for health campaigns, advanced diagnostics, AI-driven risk stratification, and innovative therapeutic approaches. | Plan does not discuss any technological opportunities. | Plan discusses some technological opportunities but does not include indicators to measure performance. | Plan discusses some technological opportunities and includes appropriate indicators. | Plan discusses all relevant technological opportunities and includes appropriate indicators. | Plan discusses all relevant technological opportunities and includes appropriate indicators comprehensively to assess them. | Plan discusses all technological opportunities, uses all indicators, and provides an analysis of the certainty, magnitude, and timing of their potential positive impact. | - High and equitable internet and smartphone penetration rates. - Widespread adoption of interoperable Electronic Health Records (EHRs) with clinical decision support systems. - Availability and integration of telehealth/digital health for patient self-management and remote cardiac rehabilitation. - Availability and promotion of cost-effective fixed-dose combination therapy (polypill). - Use of mobile coverage for public awareness campaigns - Use of novel therapies (e.g., SGLT-2 inhibitors, GLP-1 agonists, novel lipid-lowering agents) where appropriate and cost-effective. |
| **ELEMENT 6: CARDIOVASCULAR DISEASE STRATEGY** | **Description** |  |  |  | **Score** |  |  | **Example Indicators** |
| **How will the challenges identified be addressed to improve health system performance and create value in relation to CVD?** |  | **0** | **1** | **2** | **3** | **4** | **5** |  |
| **Vision** | A forward-looking statement that describes what the plan aspires to achieve in the future, grounded in a comprehensive needs assessment. Is sets out long-term goals and aspirations for the health system in relation to CVD and sets out the political commitment and governance reforms that will secure sustained uptake of the plan. | No vision statement provided. | A vision statement is provided, but it is vague or not forward-looking. | The vision statement is forward-looking, but it is not specifically descriptive of long-term goals in relation to CVD control. | The vision statement is forward-looking and descriptive of long-term goals for CVD control but lacks documented political endorsement. | The plan provides a forward-looking vision statement that is descriptive of long-term goals for CVD control and has documented high-level political endorsement. | The plan provides a forward-looking vision statement that is descriptive of long-term goals for CVD control, has documented high-level political endorsement, and is explicitly aligned with relevant international targets. | - Vision statement is forward-looking and describes long-term goals for CVD control (e.g., a nation free from the avoidable burden of CVD). - High-level political endorsement (e.g., ministerial, parliamentary) is documented, signifying commitment. - Inclusion of integrated, value-based principles (e.g., oral-systemic health integration) in the vision statement - Clarity of the plan's role in mobilizing non-health sectors |
| **Mission** | A statement that defines the plan’s actionable purpose and what it will do for the health systems, its users, citizens and society supported by a clearly defined governance structure | No mission statement provided. | A mission statement is provided, but it is unclear or does not define the plan's core purpose. | The mission statement defines a purpose but does not specify its role for key stakeholders or is not supported by a clear governance structure. | The mission statement clearly defines its purpose for key stakeholders, but the supporting governance-structure is unclear or has poorly defined roles. | The plan provides a clear mission statement that defines its purpose for key stakeholders and is supported by a clearly defined governance structure with roles and responsibilities. | The plan provides a clear mission statement that defines its purpose for key stakeholders, is supported by a defined governance structure, and is explicitly linked to strengthening primary healthcare as the foundation for its activities. | - Mission statement clearly defines the plan's purpose and what it will do for key stakeholders (patients, providers, society). - A clear governance structure (e.g., NCD commission, lead agency) is defined with roles and responsibilities to carry out the mission. |
| **Goals** | The high-level outcomes the plan aims to achieve in terms of health outcomes, financial risk protection, and user satisfaction, aligned with the mission and vision. | No goals provided. | Goals are provided, but they are vague and not aligned with the vision/mission or with specified health system outcomes. | Goals are provided and aligned with the vision/mission, but lack specific, measurable targets. | Goals are aligned with the vision/mission and have some SMART targets, but these do not comprehensively cover all outcome domains. | The plan provides goals that align with all outcome domains and have specific, measurable targets for each. | The plan provides goals that are aligned with all outcome domains, have specific and measurable targets, and explicitly address health equity by setting targets to reduce identified disparities in outcomes. | - Goals describe how the plan will achieve improved health system outcomes for CVD (as defined in Element 1). - Specific, Measurable, Achievable, Relevant, Time-bound targets are set for key outcome indicators (e.g., "Reduce premature CVD mortality by 30% by 2030"). - Establishment of high-level goals for improving patient-reported outcomes and quality of life - Establishment of high-level goals for managing key comorbidities |
| **Objectives** | How value will be created in terms of efficiency, effectiveness, equity, and responsiveness to support the attainment of the plan's goals. | No objectives provided. | Objectives are provided, but they are vague or are not clearly linked to the plan's goals. | Objectives are provided and linked to goals, but lack specific, measurable targets. | Objectives are linked to goals and have some SMART targets, but do not comprehensively cover all performance dimensions. | The plan provides objectives that cover all performance dimensions and have specific, measurable targets for each. | The plan provides objectives that cover all performance dimensions with SMART targets and are clearly and logically linked to the specific interventions and implementation strategies detailed elsewhere in the plan. | - Objectives describe how the plan will improve the efficiency, effectiveness, equity, and responsiveness of CVD services (as defined in Element 2). - Specific, Measurable, Achievable, Relevant, Time-bound targets are set for key performance objective indicators |
| **Values** | Core beliefs and principles that should guide stakeholder behaviour, decisions, and relationships when implementing the plan, reflecting the overall culture that defines actions within the health system. | No values provided. | Some values are mentioned but are not defined or explained. | Core values and principles are defined, but they are generic and not specifically applied to the context of CVD care. | Core values are defined and explicitly linked to the principles of CVD prevention and management. | The plan defines core values, links them to the principles of CVD care, and describes how these values should guide stakeholder behaviour and decision-making during implementation. | The plan defines core values, links them to CVD care, describes how they guide stakeholder behaviour, and includes a mechanism to monitor that these values are being upheld throughout the implementation and evaluation process. | - Core values and principles that guide the plan are explicitly stated. - Values are described in relation to key principles such as being patient-centered, equity-focused, evidence-based, and transparent. |
| **ELEMENT 7: GOVERNANCE AND ORGANIZATION** | **Description** |  |  |  | **Score** |  |  | **Example Indicators** |
| **What reforms are to be undertaken in terms of governance and organization of the health system to achieve the articulated strategy to improve CVD care and control?** |  | **0** | **1** | **2** | **3** | **4** | **5** |  |
| **Macro-Organization** | Key organizations within the Ministry of Health and other relevant government or quasi-governmental macro-level organizations that will be established or currently existing that assume the responsibility for overseeing the implementation and monitoring of the National CVD Plan. | No mention of the establishment of new or existing institutions or entities. | Mentions the establishment of new or existing institutions or entities but does not discuss their responsibilities. | Mentions the establishment of institutions or entities and provides a vague description of their responsibilities. | Mentions the establishment of institutions or entities and clearly discusses how they will assume responsibility for overseeing implementation and monitoring. | Mentions institutions, discusses their responsibilities, and describes the coordination mechanisms between different relevant government ministries or agencies. | The plan names a lead agency, details its responsibilities, describes inter-agency coordination mechanisms, and includes a clear organogram of the governance structure. | - A designated lead agency or unit (e.g., NCD unit within the Ministry of Health) with clear responsibility for the plan's implementation is identified. - Coordination mechanisms between different macro-level organizations (e.g., ministries of finance, education, urban planning) are described. |
| **Governance** | How the principles of accountability and transparency will be upheld concerning plan implementation, and the role of multi-sectoral collaboration, stronger alignment between ministries, professional societies, and civil society/patient advocacy groups; co-implementation, and citizen engagement – and in cases where relevant prevention of corruption, particularly around procurement processes | Does not describe how greater governance will be promoted. | Describes how greater governance will be promoted in only one of the four critical areas . | Describes how greater governance will be promoted in two of the four critical areas. | Describes how greater governance will be promoted in three of the four critical areas. | Describes how greater governance will be promoted in all four of the critical areas. | The plan describes how governance will be promoted in all four critical areas and provides specific, practical steps and responsible parties for each. | - Mechanisms for accountability and transparency in plan implementation and resource use. - Framework for multi-sectoral collaboration (e.g., with other government ministries, private sector, and NGOs). - Processes for citizen and community engagement in planning and monitoring. - Mechanisms for prevention of corruption, particularly around procurement processes. - Adoption of multidisciplinary/multi-sectoral governance boards - Mandate for public/transparent reporting of plan outcomes and costs |
| **Policy** | List and description of other relevant policies at the domestic level and resolutions or guidelines at the global level that influence the National CVD Plan. | Does not list or describe relevant policies or resolutions influencing the plan. | Lists relevant domestic or global policies but does not describe their implications for the plan. | Lists relevant domestic and global policies but does not describe their implications for the plan. | Lists relevant policies and describes some of the implications. | Lists relevant policies and describes most of the implications on the plan. | The plan lists relevant domestic and global policies and describes all implications. | - Lists relevant domestic policies (e.g., primary care strategy, national NCD plan) influencing the plan. - Lists relevant global or regional resolutions and guidelines (e.g., WHO HEARTS, WHF Roadmaps, ESC/AHA guidelines) influencing the plan. - Describes the implications of these policies on the plan in terms of (1) design, (2) implementation, (3) evaluation, and (4) maintaining political priority. |
| **Regulation** | Regulations that will be established to strengthen CVD care and control in relation to: Licensing, accreditation, certification; Quality assurance including clinical guidelines to promote effective, efficient, equitable and responsive health services; Relationship between public and private providers and payers in the health system in relation to delivery of health services for CVD and their reimbursement; Health Technology Assessment; procurement of products required for delivery of health services for CVD | Does not describe any new or strengthened regulations. | Describes new or strengthened regulations affecting one of the five critical areas. | Describes new or strengthened regulations affecting two of the five critical areas. | Describes new or strengthened regulations affecting three of the five critical areas. | Describes new or strengthened regulations affecting four of the five critical areas. | The plan describes new or strengthened regulations affecting all five of the critical areas. | - Licensing, accreditation, or certification standards for healthcare facilities providing CVD care. - Quality assurance mechanisms, including the adoption of evidence-based clinical guidelines (e.g., WHO HEARTS protocols) to promote effective and equitable services. - Regulation of public-private provider and payer relationships in the delivery of CVD services. - Processes for Health Technology Assessment for new CVD medicines and technologies. - Regulations for the procurement of products required for CVD services to ensure quality and value. - Mechanisms to ensure provider/stakeholder understanding and uptake of HTA decisions |
| **Decentralization** | Reforms implemented to change the extent of decentralization within the health system to enable plan implementation, particularly strengthening primary and community-level care delivery. | The extent of decentralization is not discussed. | Decentralization is mentioned but with no detail on which levels of the health system are affected. | The plan discusses which levels of the health system will be provided with greater decision space. | The plan discusses which levels will have greater decision space and outlines a plan for building capacity at the local level. | The plan discusses which levels will have greater decision space, outlines a capacity-building plan, and specifies accountability mechanisms. | The plan provides a detailed decentralization strategy, including decision space, capacity-building, accountability mechanisms, and dedicated funding for local-level implementation. | - Discussion of the extent of decentralization and which health system levels (national, regional, local) will be provided with greater decision-making space. - A plan for capacity-building and accountability measures for local-level actors (e.g., primary care providers, community health workers). - Defined role and integration of community health agents/workforce in CVD care delivery |
| **Strategic public private partnerships** | The design and implementation of strategic PPPs that could be used to expand funding, align incentives of key stakeholders, and promote the achievement of priority activities and outcomes in the National CVD Plan. | Does not mention public private partnerships. | Mentions PPPs but does not outline how they might be applied to support specific activities or goals of the plan. | Discusses PPPs, outlines how they will be applied to specific activities, but does not discuss the critical elements of PPP design. | Discusses using PPPs for specific activities and examines at least one of the critical elements of PPP design. | Discusses using PPPs for specific activities and examines some of the critical elements of PPP design. | The plan details how PPPs will be used for specific activities and examines all of the critical elements of PPP design to ensure their success. | - Plan to use PPPs for specific activities, objectives, or goals. - Plan to enhance government capacity to design and implement PPPs. - Plan to manage risk and reward by aligning incentives between government and private stakeholders. - Plan to build trust and transparency in PPPs. - Involvement of senior leadership in overseeing PPPs. |
| **Integration with Noncummunicable Disease Programs** | Strategies to integrate CVD control efforts with broader noncommunicable disease (NCD) prevention and control programs, leveraging shared risk factors (integrating key co-morbidities under a unified framework, and sharing health system resources to enhance efficiency and impact. | Plan does not discuss integration of CVD control with NCD programs and does not include indicators to measure performance to assess integration | Plan discusses some integration of CVD control with NCD programs but does not include indicators to measure performance to assess integration | Plan discusses some integration of CVD control with NCD programs and includes appropriate indicators to assess integration | Plan discusses comprehensive integration of CVD control with NCD programs, addressing shared risk factors and resources, and includes appropriate indicators comprehensively to assess integration | Plan discusses comprehensive integration, includes appropriate indicators comprehensively, and includes targets demonstrating performance | Plan discusses comprehensive integration, includes appropriate indicators comprehensively, includes targets demonstrating performance, and links how integration outputs, objectives, and inputs contribute to improved CVD and NCD outcomes | - Percentage of CVD prevention activities integrated with NCD programs - Number of shared surveillance systems for CVD and NCD risk factors - Percentage of health facilities offering integrated CVD and NCD services, - Funding allocated to integrated CVD-NCD initiatives as a percentage of total NCD budget. - Existence of integrated care pathways for CVD and key comorbidities |
| **ELEMENT 8: FINANCING** | **Description** |  |  |  | **Score** |  |  | **Example Indicators** |
| **What reforms are to be undertaken in terms of financing of the health system to achieve the articulated strategy to improve CVD care and control?** |  | **0** | **1** | **2** | **3** | **4** | **5** |  |
| **Cost measurement systems** | Practices and efforts to ascertain the cost of delivering health services for CVD care and control, including both direct medical costs and indirect societal costs. | No cost measurement systems are discussed. | Mentions the concept of cost but has no system in place to assess the cost of CVD services. | A cost measurement system is in place to assess either direct costs of CVD services or indirect costs to society, but not both. | A cost measurement system is in place to assess both direct costs of CVD services and indirect costs to society. | The plan details a system to assess direct and indirect costs and provides some current cost estimations for key CVD interventions. | The plan details a comprehensive system to assess direct and indirect costs, provides current cost estimations, and outlines how this data will be used for cost-effectiveness analysis and priority setting. | - Measurement of direct costs of CVD health services (e.g., cost per cardiac rehabilitation program, cost per hospital admission for AMI). - Measurement of indirect costs of CVD on society (e.g., lost productivity, informal care costs). |
| **Current financing and fiscal space** | Current level of funding allocated to health services for CVD, sources of funding, and how these sources of funding are pooled, channelled, allocated and providers remunerated for delivering health services for CVD. The plan describes current fiscal space for CVD care and control | Current spending on health services for CVD is not provided. | Current spending on health services for CVD is provided only at an aggregate level. | Current spending on CVD services is provided and disaggregated by at least one of the recommended categories. | Current spending on CVD services is provided and disaggregated by some of the recommended categories. | Current spending on CVD services is provided and disaggregated by all of the recommended categories. | The plan provides current spending data disaggregated by all categories and includes an analysis of the current fiscal space for expanding CVD funding. | - Total current spending on CVD services. - Disaggregation of spending by type of CVD (e.g., coronary artery disease, cerebrovascular disease, heart failure). - Disaggregation of spending by level of facility (primary, secondary, tertiary). - Disaggregation of spending by public vs. private sector. |
| **Proposed funding to implement plan** | The estimated funding range or detailed budget required to successfully implement the National CVD Plan. | No budget or range of funding is provided to implement the plan. | A vague or partial funding range is provided. | A clear funding range to implement the plan is provided. | A detailed budget is provided that describes the total funds required. | A detailed budget is provided that describes fund allocation to different strategic areas. | A detailed, multi-year budget is provided, allocating funds to specific activities and linking them to expected outcomes, with a clear rationale for the allocation. | - A funding range needed to implement the plan is provided. - A detailed budget is provided that allocates funds to specific activities and strategic priorities within the plan. |
| **Sources of funds** | New sources of funding, including innovative financing mechanisms, that will be designed and introduced to expand fiscal space for CVD care and control. | New sources of funding for the plan are not mentioned. | New sources of funding are mentioned in general terms. | New sources from either public or private sectors are mentioned and quantified | New sources from both public and private sectors are mentioned and quantified. | The plan identifies new public and private sources, quantifies them, and discusses innovative financing mechanisms. | The plan identifies new public, private, and innovative funding sources, quantifies them with targets for revenue generation, and provides a strategy for implementation. | - Identification of new or expanded public funding sources (e.g., earmarked health taxes on tobacco, alcohol, or sugar-sweetened beverages). - Identification of new or expanded private funding sources. - Identification of innovative financing mechanisms (e.g., blended finance, public-private partnerships). |
| **Pooling of funds** | How funds from different sources will be pooled at national or sub-national levels to purchase CVD services and reduce financial barriers for patients. | Plan does not discuss whether funds will be pooled or not. | Plan mentions that funds will be pooled but provides no additional details. | Plan discusses the pooling of funds and provides some details on the mechanism. | Plan discusses the pooling mechanism and provides details on how pooling will occur. | Plan discusses the pooling mechanism in detail and links it to another relevant policy on universal health coverage. | The plan provides a detailed description of the pooling mechanism and outlines how it will be leveraged specifically to increase access and reduce out-of-pocket costs for essential CVD care. | - Description of the fund pooling mechanisms (e.g., national health insurance fund, government general revenue). - Discussion of how pooling mechanisms will be used to improve equity, coverage, and financial protection for CVD services. |
| **Chanelling of funds** | The entities that will receive funding to implement activities prioritized by the National CVD Plan. | Plan does not discuss which entities will receive the funds. | Plan vaguely mentions entities that will receive funds. | Plan discusses which entities will receive the funds to be allocated. | Plan clearly identifies the primary entities that will receive funds. | Plan identifies all key entities at different levels of the health system that will receive funds. | The plan identifies all key entities, specifies their roles in implementation, and describes the mechanism for fund disbursement to these entities. | - Identification of the specific entities (e.g., ministries, regional health authorities, primary care networks, public hospitals) that will receive funds to implement the plan. |
| **Allocation of funds** | The amount of, and rationale for, fund allocation in relation to the specific health system resources and activities listed and described in the plan's budget, explicitly linking resources to defined outcomes and patient experience, and ensuring investment in both treatment activities and public health infrastructure. | No budget is provided or there is no mention of how funds will be allocated. | A budget is provided, but with no clear allocation to different activities or priorities. | The budget describes where funds will be allocated to high-level categories. | The budget describes where funds will be allocated to specific strategic priorities from the plan. | The budget provides a detailed allocation of funds to specific priorities and provides a rationale for the allocation. | The plan provides a detailed budget with clear allocation and rationale, and explicitly links the allocation amounts to the achievement of specific plan objectives and targets. | - The budget describes where funds will be allocated across different strategic priorities (e.g., prevention, treatment, rehabilitation, workforce). - Percentage of budget transparently linked to defined outcomes - Defined allocation for public health infrastructure |
| **Payment mechanisms for providers** | How healthcare providers will be remunerated for delivering CVD services, with an emphasis on transitioning towards value-based methods of remuneration that incentivize quality and efficiency by linking payment to health outcomes and patient experience. | There is no discussion about how providers will be remunerated. | Mentions current payment mechanisms but no discussion of reform | The merits of various payment mechanisms are discussed as options to improve efficiency, but no commitment to change is made. | The plan discusses how provider payments could change to better align with the plan's goals. | The plan commits to a specific change in provider payments to help achieve the plan's objectives. | The plan details a strategy to reform provider payments, linking specific mechanisms to specific plan objectives and targets, and includes an evaluation plan. | - Discussion of current provider payment mechanisms (e.g., fee-for-service, capitation) for CVD care. - A plan to introduce, pilot, or scale up value-based or performance-based payment mechanisms to improve care quality and outcomes. - Proportion of CVD programs financed under Value-Based Health Care principles |
| **Payment mechanisms for capital investments** | How procurement processes will be executed with an emphasis on transitioning towards value-based methods of procurement. Value-based procurement refers to the purchasing of healthcare products in a manner that prioritises value instead of focusing only the lowest price. Value is measured in terms of efficiency, effectiveness, equity and responsiveness of health services and outcomes produced. Value-based procurement is characterised by greater collaboration between vendors and providers, balancing of risk and reward and considering how product design influences delivery of health services | Procurement of capital-intensive investments is not discussed. | Procurement of capital-intensive investments is discussed, but with no mention of value-based approaches. | Value-based procurement is identified as an option to explore for capital-intensive investments. | The plan states an intention to use value-based procurement for capital-intensive investments. | The plan discusses how value-based procurement will be applied to specific capital-intensive investments. | The plan provides a detailed strategy for using value-based procurement for specific investments, includes these items in the budget, and defines metrics for assessing value. | - Discussion of procurement processes for capital-intensive investments such as new facilities, cath labs, or imaging equipment. - Identification of value-based procurement as an option to explore or implement for these investments. |
| **Resource Mobilisation Strategies** | Approaches to secure and sustain financial and non-financial resources for NCVDCP implementation, including domestic funding, international partnerships, private sector contributions, and innovative financing mechanisms , to ensure long-term program viability | Plan does not discuss any resource mobilization strategies for NCVDCP implementation and does not include indicators to measure performance to assess these strategies | Plan discusses some resource mobilization strategies but does not include indicators to measure performance to assess these strategies | Plan discusses some resource mobilization strategies and includes appropriate indicators to assess these strategies | Plan discusses comprehensive resource mobilization strategies, including domestic, international, and private sector sources, and includes appropriate indicators comprehensively to assess these strategies | Plan discusses comprehensive resource mobilization strategies, includes appropriate indicators comprehensively, and includes targets demonstrating performance | Plan discusses comprehensive resource mobilization strategies, includes appropriate indicators comprehensively, includes targets demonstrating performance, and links how mobilization outputs, objectives, and inputs contribute to sustained NCVDCP implementation | - Percentage of NCVDCP budget funded by domestic sources - Amount of international donor funding secured for CVD control - Number of private sector partnerships established for NCVDCP funding - Percentage of NCVDCP activities supported by innovative financing mechanisms |
| **ELEMENT 9: RESOURCE MANAGEMENT** | **Description** |  |  |  | **Score** |  |  | **Example Indicators** |
| **What reforms are to be undertaken in terms of resource management of the health system to achieve the articulated strategy to improve CVD care and control?** |  | **0** | **1** | **2** | **3** | **4** | **5** |  |
| **Human Resources** | The training, recruitment, interprofessional integration, equitable distribution, and deployment of a qualified health workforce to prevent, manage, and rehabilitate patients with cardiovascular conditions, with particular attention to remote and vulnerable populations. | Additional human resources are not mentioned. | Additional human resources are mentioned but are not quantified. | Additional human resources are mentioned and quantified, but no targets for increases are provided. | Additional human resources are quantified with targets, but the targets are not disaggregated by type of healthcare worker. | Additional human resources are quantified with targets and are disaggregated by type of healthcare worker. | The plan quantifies human resources with targets disaggregated by type of worker and outlines a clear strategy for their training, recruitment, and deployment to underserved areas. | - Physicians per 100,000 population - Number of cardiologists per 100,000 population - Number of cardiac surgeons per 100,000 population - Number of primary care providers trained in CVD management - Health workforce density and distribution (urban vs. rural) - Equity index for distribution of human resources across regions |
| **Infrastructure** | Facilities, equipment and devices, and the maintenance of these facilities and equipment | Additional infrastructure needs are not mentioned. | Additional infrastructure is mentioned but is not quantified using any of the suggested indicators. | Additional infrastructure is mentioned and quantified, but no targets for expansion are provided. | Additional infrastructure is quantified with targets, but the targets are not disaggregated by type of infrastructure. | Additional infrastructure is quantified with targets and is disaggregated by type of infrastructure. | The plan quantifies infrastructure with targets disaggregated by type and includes a strategy for equitable geographic deployment to address gaps in access. | - Hospital beds per 100,000 population - Cardiac Care Unit (CCU) / Intensive Care Unit (ICU) beds per 100,000 population - Number of facilities with cardiac catheterization (cath lab) capabilities - Number of dedicated cardiac rehabilitation facilities |
| **Pharmaceuticals and medical supplies** | The essential medicines, therapeutics, and supplies used for the prevention, diagnosis, and treatment of cardiovascular disease. | Additional pharmaceutical needs are not mentioned. | Additional pharmaceuticals are mentioned but are not quantified or specified. | Additional pharmaceuticals are mentioned and quantified, but no targets for availability or affordability are provided. | Additional pharmaceuticals are quantified with targets, but the targets are not disaggregated by type of product. | Additional pharmaceuticals are quantified with targets and are disaggregated by type of product. | The plan quantifies essential pharmaceuticals with targets for availability and affordability, disaggregates them by type, and includes a strategy for ensuring access, such as inclusion in the national essential medicines list. | - National availability and affordability of: aspirin, statins, beta-blockers - ACE inhibitors/ARBs - P2Y12 inhibitors, fixed-dose combinations (polypill), and influenza vaccine for high-risk patients. |
| **Information technology and data systems** | Encompass the infrastructure and processes required for data systems... to inform outcome measurements, clinical decision-making, research, innovation, and health policy and planning, including the use of electronic health records, artificial intelligence, and telemedicine to support data-driven resource allocation, optimize workforce deployment, and expand care coverage. | Additional information technology needs are not mentioned. | Information technology and data systems are mentioned but are not quantified. | Additional IT and data systems are mentioned and quantified, but no targets for expansion or implementation are provided. | Additional IT and data systems are quantified with targets, but the targets are not disaggregated by type of system. | Additional IT and data systems are quantified with targets and are disaggregated by type. | The plan quantifies IT and data systems with targets disaggregated by type and includes a strategy for implementation, interoperability, and ensuring data privacy and security. | - Proportion of primary care practices using an Electronic Health Record. - Presence of a national patient registry for key CVD events (e.g., AMI, stroke). - Use of clinical decision support systems for CVD risk management in primary care. - Availability of telehealth/m-health platforms for patient management and cardiac rehabilitation. - Percentage of health facilities with integrated digital systems for CVD monitoring |
| **Supply Chain Management** | The procurement, storage, distribution, inventory management, and monitoring of essential medical products, equipment, and pharmaceuticals necessary for CVD prevention, diagnosis, and treatment. | Strengthening of supply chain management is not mentioned. | Mentions only one of the four critical aspects of supply chain management. | Mentions some of the four critical aspects of supply chain management. | Mentions all four of the critical aspects of supply chain management. | The plan discusses strengthening all four aspects and outlines a strategy for doing so. | The plan details a comprehensive strategy to strengthen all four aspects of the supply chain, including the use of technology for monitoring and specific actions to reduce stockouts of essential CVD medicines. | - A plan is in place to strengthen the supply chain for essential CVD medicines and supplies across four critical aspects: (1) Procurement, (2) Distribution, (3) Storage, (4) Inventory management. |
| **Research** | Efforts designed to generate new knowledge in relation to cardiovascular disease care and control that is relevant to the national context, including clinical, health systems, and implementation research. | No mention of additional research needs. | Discusses the need for additional research but does not provide any details on priorities or structures. | Discusses the need for additional research and identifies broad priority areas. | Discusses research needs, identifies priorities, and provides targets for research centres or funding. | The plan discusses research needs, provides targets, and outlines a clear national CVD research agenda. | The plan outlines a national CVD research agenda with clear priorities, commits to specific funding mechanisms, and includes a strategy for translating research findings into policy and practice. | - Number of national CVD research centres or networks. - Level of national funding or commitment to funding for CVD research. - A plan for comparative effectiveness or implementation research to address local disparities and challenges. |
| **Innovation ecosystem** | The institutionalization of "push" and "pull" strategies and policy changes that promote emergent and driven innovation to encourage the design, development, implementation, and scale-up of new solutions for CVD care. | No mention of the need to develop an innovation ecosystem for CVD. | Discusses the need to develop an innovation ecosystem but does not provide any additional details. | Discusses the need to develop an innovation ecosystem and provides additional details on priorities for innovation. | The plan details priorities for innovation and discusses how these could be achieved. | The plan details priorities for innovation, how they could be achieved, and how they could be assessed or evaluated. | The plan details a comprehensive strategy for fostering a CVD innovation ecosystem, including priorities, implementation mechanisms, and a framework for assessment and scaling up successful innovations. | - Existence of policies to promote innovation in CVD care (e.g., in digital health, new care models). - Public-private partnerships for research and development of new CVD technologies or therapies. - Mechanisms for the assessment and scaling up of proven innovations |
| **Capacity Building for Health Professionals** | Strategies and initiatives to enhance the skills, knowledge, and availability of health professionals involved in CVD control, including continuous / lifelong training and retraining programs for prevention, early detection, diagnosis, treatment, and palliative care, as well as in quality, safety, and the use of digital health tools, to strengthen the workforce and improve service delivery | Plan does not discuss any capacity building initiatives for health professionals involved in CVD control and does not include indicators to measure performance to assess these initiatives | Plan discusses some capacity building initiatives for health professionals but does not include indicators to measure performance to assess these initiatives | Plan discusses some capacity building initiatives for health professionals and includes appropriate indicators to assess these initiatives | Plan discusses comprehensive capacity building initiatives for health professionals across all CVD control areas and includes appropriate indicators comprehensively to assess these initiatives | Plan discusses comprehensive capacity building initiatives, includes appropriate indicators comprehensively, and includes targets demonstrating performance | Plan discusses comprehensive capacity building initiatives, includes appropriate indicators comprehensively, includes targets demonstrating performance, and links how training outputs, objectives, and inputs contribute to improved CVD control outcomes | - Number of health professionals trained in CVD prevention and control per 100,000 population - Percentage of primary care providers trained in early detection of CVD - Number of specialized CVD training programs established. - Proportion of health professionals trained in updated CVD protocols |
| **Education Initiatives** | How the health system is currently performing in relation to CVD, assessed in terms of educational initiatives that promote CVD awareness, prevention, and early detection among the population, including health literacy programs, school-based education, and community outreach efforts, as well as the training of community partners to support awareness and prevention. | Plan does not discuss any educational initiatives for CVD awareness, prevention, or health literacy. | Plan discusses some educational initiatives (e.g., a national campaign) but does not use specific indicators to measure or assess them. | Plan discusses some educational initiatives (e.g., health literacy, school programs) and uses appropriate indicators to assess them. | Plan discusses all key educational initiatives (awareness, literacy, school, community partners) and uses appropriate indicators comprehensively to assess them. | Plan discusses all key educational initiatives, uses appropriate indicators comprehensively, and provides national baseline data on the reach and availability of these programs. | Plan discusses all key educational initiatives, uses appropriate indicators comprehensively with baseline data, and includes specific, measurable targets for improving health literacy and program coverage. | - Existence of national CVD awareness campaigns - Integration of health literacy programs into primary care - Availability of school-based health education programs - Existence of programs to train non-clinical partners (e.g., NGOs, patient survivors) in awareness-raising |
| **ELEMENT 10: HEALTH SERVICES** | **Description** |  |  |  | **Score** |  |  | **Example Indicators** |
| **What reforms are to be undertaken in terms of the delivery of health services to achieve the articulated strategy to improve CVD care and control?** |  | **0** | **1** | **2** | **3** | **4** | **5** |  |
| **Public Health Services (Health promotion)** | Primary prevention interventions focusing on the social and environmental determinants of CVD that empower individuals to lead healthier lifestyles in relation to CVD risk factors: tobacco use, harmful alcohol consumption, unhealthy diets, and physical inactivity and ensuring these services are equitably delivered and accessible to all, including vulnerable populations. | No health promotion interventions are described. | Interventions are mentioned for only 1-2 risk factors with no detail. | Interventions are described for most key risk factors. | Interventions are described for all key risk factors with clear implementation plans. | As score 3, but also includes specific, measurable targets for public reach or engagement. | As score 4 and includes a mechanism for monitoring and evaluating the effectiveness of the interventions. | - Number and reach of national public health campaigns focused on CVD risk factors. - Availability and accessibility of community-based lifestyle modification programs (e.g., smoking cessation, healthy diet support). - Implementation of public health policies that create healthy environments (e.g., urban planning for walkability). - Percentage of population covered by primary prevention programs (e.g., smoking cessation, nutrition counselling) |
| **Public Health Services (Health protection)** | Primary prevention interventions focusing on environmental and policy determinants to protect individuals and populations from CVD. This relates to risk factors such as tobacco use, unhealthy diets, and air pollution and ensuring these policies are equitably applied. | No health protection policies are described. | Policies are mentioned for at least one risk factor but lack detail or commitment. | Clear policies are described for several key risk factors . | A comprehensive suite of policies is described for all key risk factors. | As score 3, with clear evidence of strong enforcement mechanisms. | As score 4, with a system in place to monitor the population-level impact of the policies. | - Existence and strength of tobacco control legislation (e.g., taxes, smoke-free policies). - Regulations on food composition and labelling (e.g., mandatory limits on sodium or trans-fats). - Restrictions on the marketing of unhealthy foods and beverages to children. |
| **Personal Healthcare Services (Disease Prevention)** | Interventions aimed at preventing CVD and promoting early detection through screening for key risk factors. Common risk factors screened for include clinical factors as well as healthy living behaviours. Testing interventions may be clinical, laboratory, or questionnaire based. These services must be equitably accessible to all, including vulnerable populations. | No screening interventions are described. | Screening for at least one major risk factor is mentioned but without a systematic approach. | Systematic screening programs are described for the 3 critical risk factors but lack detail on target populations or frequency. | Screening programs for the critical risk factors are described with clear target populations and recommended frequencies. | As score 3, and a systematic approach for calculating and acting on total cardiovascular risk is also included. | As score 4, with specific, measurable coverage targets and a clear plan for ensuring equitable access to screening. | - Percentage of the eligible adult population screened for total CVD risk using a nationally endorsed tool. - Percentage of hypertensive patients with controlled blood pressure (<140/90 mmHg). - Percentage of high-risk patients with controlled LDL-cholesterol according to guidelines. - Rate of early detection and timely referral of CVD cases from primary care |
| **Personal Healthcare Services (Diagnosis)** | The personal healthcare services to be delivered to enhance CVD diagnosis. These services include diagnostic tools, imaging, and laboratory medicine. These services must be equitably accessible to all, including vulnerable populations. | No diagnostic interventions or pathways are described. | Some diagnostic tools are mentioned, but without organised pathways for their use. | Organised pathways for diagnosis are described for some key CVD conditions. | Clear diagnostic pathways are described for all major acute and chronic CVD presentations. | As score 3, with defined targets for key performance indicators. | As score 4 and specifies how diagnostic services will be organised and funded to ensure equitable access. | - Availability of ECG and cardiac biomarker testing in primary care and emergency settings. - Access to non-invasive cardiac imaging (e.g., echocardiography, stress testing). - Defined clinical pathways and wait-time targets for timely diagnosis of acute coronary syndromes and stroke. |
| **Personal Healthcare Services (Treatment)** | The personal healthcare services to be delivered to enhance CVD treatment. These services include medical therapies, procedures, and surgery, among others. These services must be equitably accessible to all, including vulnerable populations. | No treatment interventions are described. | Some treatments are mentioned but without clear context or evidence-based pathways. | Evidence-based treatments for some major CVD conditions are described. | A comprehensive plan for providing evidence-based medical and procedural treatments across the care continuum is described. | As score 3, with specific targets for treatment quality. | As score 4 and includes strategies to ensure equitable and affordable access to essential treatments. | - Proportion of eligible patients prescribed appropriate secondary prevention medications (e.g., antiplatelets, statins). - Number of percutaneous coronary interventions (PCIs) per 100,000 population. - National availability and affordability of essential CVD medicines. |
| **Personal Healthcare Services (Palliation and rehabilitative care)** | The personal healthcare services to be delivered to enhance rehabilitation and palliation for individuals with CVD. These services must be equitably accessible to all, including vulnerable populations. | No rehabilitative or palliative services are described. | Cardiac rehabilitation or palliative care is mentioned generally without commitment. | A plan for providing cardiac rehabilitation is described, but a plan for palliative care is missing. | Plans for providing both cardiac rehabilitation and palliative care for advanced CVD are described. | As score 3, with specific targets for service uptake, completion, or availability. | As score 4 and includes strategies to overcome access barriers. | - Uptake and completion rates for cardiac rehabilitation among eligible patients. - Number and availability of cardiac rehabilitation programs (including home-based/digital models) per 100,000 eligible patients. - Availability of specialized palliative care services for patients with end-stage heart failure. |
| **Provider Value Enhancement** | Measures to be taken by providers to improve value in the delivery of health services. Value is measured in terms of the efficiency, effectiveness, equity and responsiveness of health services for CVD care and control. This includes strengthening holistic, patient-centred approaches through integrated multidisciplinary teams. Other measures include: (Electronic Health Records, (Cost measurement systems; (Outcomes measurement systems; (Benchmarking; (Integrated care pathways; (integrated provider networks; (value-based procurement; (value-based payment models, multi-disciplinary collaborative care teams. | No measures to enhance provider value are specified. | Mentions the concept of value or a few general measures without detail. | Describes plans to implement several distinct value-enhancement measures. | Describes plans to implement a majority of distinct value-enhancement measures. | As score 3, with clear implementation plans and metrics to assess progress for each measure. | As score 4, and provides a comprehensive, integrated strategy showing how these measures will work together to improve the overall value of CVD care. | - Proportion of providers using interoperable Electronic Health Records with clinical decision support. - Implementation of multidisciplinary, team-based care models. - Use of value-based payment models (e.g., pay-for-performance for hypertension control). - Number and proportion of health facilities offering integrated multidisciplinary CVD services |
| **ELEMENT 11: MONITORING AND EVALUATION** | **Description** |  |  |  | **Score** |  |  | **Example Indicators** |
| **How will the plan be implemented, measured, stakeholder alignment and sustained change management be achieved?** |  | **0** | **1** | **2** | **3** | **4** | **5** |  |
| **Monitoring and Evaluation framework** | How the achievement of the plan is evaluated in relation to its inputs, activities, outputs, and outcomes. A comprehensive M&E plan should include defined activities, expected outcomes, indicators with targets, and timelines. A comprehensive M&E plan should be closely aligned with measurable and time-bound objectives and include defined activities, expected outcomes, indicators with targets, and timelines. It should integrate digital health tools for continuous monitoring of progress, disparities, and resource allocation. The M&E framework should ensure a balanced focus on both prevention and management goals and may involve independent or neutral individuals for monitoring oversight. | A monitoring and evaluation plan is not provided. | An M&E plan is mentioned, but it is missing most critical aspects. | The M&E plan provided is missing some critical aspects. | The M&E plan provided includes all critical aspects but lacks specific detail or clear linkages between them. | The plan provides a complete M&E framework with all critical aspects clearly defined and linked. | The plan provides a complete M&E framework and details how the results will be used for continuous quality improvement and to inform future planning cycles. | - A list of plan activities is provided. - Expected outputs and outcomes are linked to activities. - Indicators and targets are defined to measure progress against outputs and outcomes. - Timelines, deadlines, and milestones are established to guide implementation. - Responsibilities for data collection, analysis, and reporting are clearly assigned. - Existence of equity indicators (e.g., CVD outcomes disaggregated by age, gender, geography, socioeconomic status) - Inclusion of key process metrics (e.g., % of primary care facilities implementing guidelines; patient adherence rates) - Inclusion of Patient-Reported Outcome Measures and Experience Measures in the M&E dashboard - Defined mechanisms for tracking multidisciplinary team engagement |
| **Change management** | How change management will be achieved and the approach that will be pursued to promote transparency, accountability, and the adoption of new practices by stakeholders. This requires clear accountability mechanisms, strong stakeholder engagement, and continuous capacity-building at all levels of the health system to ensure the adoption of new practices. | A change management approach is not mentioned. | A change management approach is mentioned, but critical aspects are not described. | A change management approach is mentioned with a description of some of the critical aspects. | A change management approach is mentioned with a description of most of the critical aspects. | The plan outlines a comprehensive change management approach covering all critical aspects. | The plan outlines a comprehensive change management approach and provides a detailed implementation plan for each aspect, with assigned responsibilities and timelines. | - Assessment of stakeholder readiness, potential resistance, and drivers for change. - A strategy for leadership engagement to champion the plan. - A communication plan to keep all stakeholders informed throughout implementation. - A training and capacity-building plan for the health workforce to adopt new protocols and practices. - Inclusion of a continuous capacity-building plan to support change |
| **Risk and Mitigation Strategies** | The identification and analysis of major risks that could influence the implementation of the cardiovascular disease control plan including political risks, financial, and operational risks, and the mitigation measures that will be pursued to better manage these risks. | Risks and mitigation measures are not specified. | Risks are identified, but no analysis or mitigation measures are provided. | Risks are identified and analysed in terms of likelihood or impact, but mitigation measures are not specified. | Risks are identified and analysed, and general mitigation measures are provided. | Risks are identified and analysed, and specific mitigation measures are provided for each key risk. | The plan provides a detailed risk register that identifies and analyses risks, provides specific mitigation measures, and assigns responsibility for monitoring and acting on each risk. | - Identification of key implementation risks (e.g., funding shortfalls, political changes, supply chain disruptions, low stakeholder buy-in). - Analysis of risks in terms of their likelihood and potential impact on the plan's success. - Development of specific, actionable mitigation strategies for high-priority risks. |
| **Stakeholder engagement** | The expectations, responsibilities, and plan for engaging key stakeholders in the implementation of the plan, including other government ministries, sub-national governments, the private sector, non-governmental organizations, professional societies, and health professionals themselves. | Stakeholder engagement is not discussed. | Some stakeholders are identified, but their roles, responsibilities, and engagement are not discussed. | Some expectations, responsibilities, and engagement plans for key stakeholders are discussed. | Most key stakeholders are identified and their expectations, responsibilities, and engagement plans are discussed. | The plan discusses engagement for all key stakeholder groups, including government, private sector, and civil society, and provides targets to assess the progress of engagement. | The plan provides a comprehensive stakeholder engagement strategy with defined roles, responsibilities, targets, and links these engagement activities to the plan's governance and accountability structures. | - A map of key stakeholders is provided (e.g., government, professional societies, patient advocacy groups, private sector). - Roles and responsibilities for each stakeholder group in plan implementation are defined. - A plan for ongoing communication and engagement with all stakeholder groups is described. - A specific plan for engaging patients, families, and communities is included, emphasizing their role in co-design and governance. |
