# Supplementary material for: A global analysis of national cardiovascular disease control plans using a multi-agent artificial intelligence model
Source: PLOS Digit Health. 2026 Jun 1;5(6):e0001447. doi: 10.1371/journal.pdig.0001447 (PMC13225395; doi:10.1371/journal.pdig.0001447)
Supplement: S2 Table — (DOCX) [file pdig.0001447.s007.docx]

**S2: Sensitivity Analysis of Scoring Aggregation Methods**

| **Method** | **Global Median** | **Global Mean** |
| --- | --- | --- |
| Sub-element-equal, median (baseline) | 1.00 | 0.82 |
| Sub-element-equal, mean | 1.30 | 1.33 |
| Element-equal, mean | 1.18 | 1.22 |
| Element-equal, median | 1.05 | 1.14 |
