# Supplementary material for: A global analysis of national cardiovascular disease control plans using a multi-agent artificial intelligence model
Source: PLOS Digit Health. 2026 Jun 1;5(6):e0001447. doi: 10.1371/journal.pdig.0001447 (PMC13225395; doi:10.1371/journal.pdig.0001447)
Supplement: S3 Table — (DOCX) [file pdig.0001447.s008.docx]

**S3: Large Disagreements Between LLM and Human Reviewer Scores (|difference| ≥ 3)**

| **Country** | **Framework Element** | **LLM Score** | **Human Reviewer Score** | **Difference** |
| --- | --- | --- | --- | --- |
| **Ghana** | Element 7: Governance and Organization | 5.0 | 0.0 | 5.0 |
| **Myanmar** | Element 8: Financing | 5.0 | 0.0 | 5.0 |
| **United Kingdom** | Element 6: Cardiovascular Disease Strategy | 5.0 | 0.0 | 5.0 |
| **United States** | Element 6: Cardiovascular Disease Strategy | 5.0 | 0.0 | 5.0 |
| **United Kingdom** | Element 7: Governance and Organization | 5.0 | 0.0 | 5.0 |
| **Ghana** | Element 6: Cardiovascular Disease Strategy | 5.0 | 0.0 | 5.0 |
| **Turkey** | Element 9: Resource Management | 5.0 | 0.0 | 5.0 |
| **Myanmar** | Element 6: Cardiovascular Disease Strategy | 4.0 | 0.0 | 4.0 |
| **Ghana** | Element 7: Governance and Organization | 4.0 | 0.0 | 4.0 |
| **Ghana** | Element 8: Financing | 4.0 | 0.0 | 4.0 |
| **Turkey** | Element 11: Monitoring and Evaluation | 4.0 | 0.0 | 4.0 |
| **Turkey** | Element 7: Governance and Organization | 4.0 | 0.0 | 4.0 |
| **Ghana** | Element 6: Cardiovascular Disease Strategy | 4.0 | 0.0 | 4.0 |
| **Ghana** | Element 6: Cardiovascular Disease Strategy | 4.0 | 0.0 | 4.0 |
| **Ghana** | Element 9: Resource Management | 4.0 | 0.0 | 4.0 |
| **United States** | Element 10: Health Services | 4.0 | 0.0 | 4.0 |
| **Ghana** | Element 10: Health Services | 4.0 | 0.0 | 4.0 |
| **United States** | Element 8: Financing | 4.0 | 0.0 | 4.0 |
| **United Kingdom** | Element 7: Governance and Organization | 4.0 | 0.0 | 4.0 |
| **United Kingdom** | Element 6: Cardiovascular Disease Strategy | 4.0 | 0.0 | 4.0 |
| **United States** | Element 7: Governance and Organization | 4.0 | 0.0 | 4.0 |
| **India** | Element 8: Financing | 4.0 | 0.0 | 4.0 |
| **India** | Element 8: Financing | 4.0 | 0.0 | 4.0 |
| **India** | Element 8: Financing | 4.0 | 0.0 | 4.0 |
| **Myanmar** | Element 7: Governance and Organization | 4.0 | 0.0 | 4.0 |
| **Myanmar** | Element 8: Financing | 4.0 | 0.0 | 4.0 |
| **Myanmar** | Element 8: Financing | 4.0 | 0.0 | 4.0 |
| **United Kingdom** | Element 8: Financing | 4.0 | 0.0 | 4.0 |
| **United Kingdom** | Element 8: Financing | 4.0 | 0.0 | 4.0 |
| **Turkey** | Element 6: Cardiovascular Disease Strategy | 4.0 | 0.0 | 4.0 |
| **Myanmar** | Element 11: Monitoring and Evaluation | 4.0 | 0.0 | 4.0 |
| **Turkey** | Element 6: Cardiovascular Disease Strategy | 5.0 | 1.0 | 4.0 |
| **United States** | Element 7: Governance and Organization | 4.0 | 0.0 | 4.0 |
| **India** | Element 7: Governance and Organization | 5.0 | 1.0 | 4.0 |
| **India** | Element 8: Financing | 3.0 | 0.0 | 3.0 |
| **India** | Element 8: Financing | 3.0 | 0.0 | 3.0 |
| **India** | Element 8: Financing | 3.0 | 0.0 | 3.0 |
| **Turkey** | Element 10: Health Services | 3.0 | 0.0 | 3.0 |
| **Turkey** | Element 7: Governance and Organization | 4.0 | 1.0 | 3.0 |
| **Myanmar** | Element 4: Health System Threats | 4.0 | 1.0 | 3.0 |
| **United Kingdom** | Element 8: Financing | 3.0 | 0.0 | 3.0 |
| **Ghana** | Element 1: Health System Performance Outcomes | 3.0 | 0.0 | 3.0 |
| **Myanmar** | Element 6: Cardiovascular Disease Strategy | 4.0 | 1.0 | 3.0 |
| **Myanmar** | Element 7: Governance and Organization | 4.0 | 1.0 | 3.0 |
| **Myanmar** | Element 8: Financing | 3.0 | 0.0 | 3.0 |
| **Myanmar** | Element 8: Financing | 3.0 | 0.0 | 3.0 |
| **Myanmar** | Element 9: Resource Management | 4.0 | 1.0 | 3.0 |
| **Myanmar** | Element 10: Health Services | 4.0 | 1.0 | 3.0 |
| **Myanmar** | Element 10: Health Services | 3.0 | 0.0 | 3.0 |
| **United States** | Element 7: Governance and Organization | 3.0 | 0.0 | 3.0 |
| **United States** | Element 7: Governance and Organization | 5.0 | 2.0 | 3.0 |
| **United States** | Element 8: Financing | 3.0 | 0.0 | 3.0 |
| **United States** | Element 8: Financing | 3.0 | 0.0 | 3.0 |
| **United States** | Element 8: Financing | 3.0 | 0.0 | 3.0 |
| **United States** | Element 8: Financing | 3.0 | 0.0 | 3.0 |
| **United States** | Element 8: Financing | 3.0 | 0.0 | 3.0 |
| **Ghana** | Element 6: Cardiovascular Disease Strategy | 3.0 | 0.0 | 3.0 |
| **Ghana** | Element 8: Financing | 3.0 | 0.0 | 3.0 |
| **Turkey** | Element 9: Resource Management | 0.0 | 3.0 | -3.0 |
| **United Kingdom** | Element 9: Resource Management | 0.0 | 3.0 | -3.0 |
| **United Kingdom** | Element 7: Governance and Organization | 0.0 | 3.0 | -3.0 |
| **Turkey** | Element 3: Health System Performance Outputs | 0.0 | 3.0 | -3.0 |
| **Turkey** | Element 11: Monitoring and Evaluation | 0.0 | 3.0 | -3.0 |
| **India** | Element 10: Health Services | 0.0 | 3.0 | -3.0 |
| **United Kingdom** | Element 9: Resource Management | 0.0 | 3.0 | -3.0 |
| **India** | Element 10: Health Services | 0.0 | 3.0 | -3.0 |
| **Myanmar** | Element 9: Resource Management | 1.0 | 4.0 | -3.0 |
| **India** | Element 9: Resource Management | 0.0 | 3.0 | -3.0 |
| **India** | Element 7: Governance and Organization | 0.0 | 3.0 | -3.0 |
| **India** | Element 6: Cardiovascular Disease Strategy | 0.0 | 3.0 | -3.0 |
| **India** | Element 9: Resource Management | 0.0 | 3.0 | -3.0 |
| **India** | Element 3: Health System Performance Outputs | 0.0 | 3.0 | -3.0 |
| **Turkey** | Element 10: Health Services | 0.0 | 3.0 | -3.0 |
| **India** | Element 11: Monitoring and Evaluation | 0.0 | 3.0 | -3.0 |
| **India** | Element 11: Monitoring and Evaluation | 0.0 | 3.0 | -3.0 |
| **Ghana** | Element 2: Health System Performance Objectives | 1.0 | 5.0 | -4.0 |
| **India** | Element 9: Resource Management | 0.0 | 4.0 | -4.0 |
| **Turkey** | Element 10: Health Services | 0.0 | 4.0 | -4.0 |
| **India** | Element 3: Health System Performance Outputs | 0.0 | 4.0 | -4.0 |
| **India** | Element 4: Health System Threats | 0.0 | 4.0 | -4.0 |
| **India** | Element 7: Governance and Organization | 0.0 | 4.0 | -4.0 |
| **Turkey** | Element 4: Health System Threats | 0.0 | 4.0 | -4.0 |
| **India** | Element 9: Resource Management | 0.0 | 4.0 | -4.0 |
| **India** | Element 11: Monitoring and Evaluation | 0.0 | 4.0 | -4.0 |
