# Supplementary material for: A global analysis of national cardiovascular disease control plans using a multi-agent artificial intelligence model
Source: PLOS Digit Health. 2026 Jun 1;5(6):e0001447. doi: 10.1371/journal.pdig.0001447 (PMC13225395; doi:10.1371/journal.pdig.0001447)
Supplement: Attachment — Submitted filename: PDIG-D-26-00060_reviewer_JULIAN_BORGES.pdf [file pdig.0001447.s009.pdf]

A global analysis of national cardiovascular disease control plans using a multi agent artificial intelligence model

PDIG-D-26-00060_reviewer_JULIAN_BORGES

Summary

This manuscript presents a global benchmarking analysis of standalone national cardiovascular disease control plans. The authors identify 44 plans, develop an adapted health systems planning framework comprising 11 elements and 69 sub elements, and validate the framework through an international expert consensus process. Plans are assessed using a structured multi stage computational workflow, with blinded human review on a subset of six countries used for concordance assessment.

The primary finding is that most plans articulate strategic direction and governance structures but lack operational specificity, particularly in contextual analysis, baseline performance assessment, measurable objectives, financing detail, and implementation planning. The manuscript addresses an important policy gap, is clearly written, and is generally aligned with the scope of the journal. The principal limitations relate to interpretation of validation results, statistical treatment of ordinal scores, and robustness of aggregation choices.

Major issues

1. Interpretation and support of validation results

The validation analysis reports moderate exact and near agreement between computational scoring and human review but weak correlation and very low weighted agreement. While these results are transparently reported, their interpretation remains insufficiently developed. As presented, readers may have difficulty understanding the conditions under which the approach is reliable and the specific limitations of the scoring system.

Recommendation:

- State explicitly, in the Abstract, Results, and Discussion, that the approach is validated for structural benchmarking of documented plan content rather than for fine grained ordinal scoring equivalent to expert judgment.
- Provide at least one diagnostic analysis characterizing disagreement structure, such as element level signed differences between computational and human scores or a collapsed low, medium, high score agreement table.

- Report human reviewer inter rater agreement if available. If not assessed, this should be stated explicitly and discussed as a limitation of the reference standard.
- Clarify whether observed disagreement reflects systematic generosity, score compression, or element specific limitations.

---

2. Treatment of ordinal scores as interval data

Sub element scores on a 0 to 5 rubric are analyzed as numeric quantities using Pearson correlation, arithmetic differences, and aggregated summaries. This implicitly assumes interval scale properties that are not justified for rubric based ordinal assessments and complicates interpretation of validation statistics.

Recommendation:

- Justify the numeric treatment of rubric scores or revise the emphasis of validation analyses toward ordinal appropriate summaries.
- Replace or supplement Pearson correlation with rank based or ordinal focused diagnostics.
- Clarify that reported numerical differences represent heuristic indicators of directional bias rather than precise quantitative distances.

---

3. Aggregation logic and robustness of conclusions

Element level and overall scores are derived from aggregation across multiple sub elements using an implicit equal weighting scheme. The manuscript does not assess whether key findings are robust to reasonable alternative aggregation choices.

Recommendation:

- Provide a brief sensitivity analysis demonstrating that the principal conclusions persist under alternative aggregation approaches, such as mean versus median aggregation or equal weighting by element rather than by sub element.
- Clarify the rationale for the chosen weighting scheme and define what the total score represents conceptually.

---

4. Interpretation of regional and income group comparisons

The sample is unevenly distributed across regions and income groups, with small counts in several strata. While limitations are acknowledged, some language may still be interpreted as comparative performance assessment rather than descriptive benchmarking of available documents.

Recommendation:

- Strengthen language throughout to emphasize that regional and income group analyses are descriptive and exploratory.
- Avoid phrasing that implies performance ranking or causal interpretation.
- Add a brief reminder near stratified results that small subgroup sizes limit precision and generalizability.

Minor issues

1. Terminology and labeling consistency
   One table heading retains language related to cancer control rather than cardiovascular disease control and should be corrected.
2. Clarification of score meaning
   Add a concise statement clarifying that non zero scores reflect documented presence and specificity within plans, not adequacy, effectiveness, or implementation success.
3. Reproducibility information
   Provide minimal computational reproducibility metadata, including model version identifiers and the availability of scoring schemas, prompts, or structured output formats.
4. Multilingual and document heterogeneity
   Briefly describe whether scoring reliability varied by language or document format and how ingestion and validation steps addressed this variability.
5. Contextual elements scoring criteria
   Clarify whether narrative contextual discussion without explicit headings was eligible for scoring and how implicit content was handled, given the very low scores for threats and opportunities.

Other comments

The validated framework represents a substantial contribution and should be emphasized as a reusable policy instrument independent of the computational workflow. The manuscript is strongest when it positions the computational system as a scalable tool for structural benchmarking and prioritization for expert review, rather than as a substitute for qualitative policy assessment.

Figures and tables: Clearly presented and correctly labeled

Yes overall. Figures and tables are clear and interpretable, subject to correction of the noted labeling inconsistency.

Methods: Sufficiently detailed?

Largely yes for conceptual understanding. Additional detail is recommended for computational reproducibility and validation interpretation.

Study design: Appropriate for the question?

Yes. A cross sectional structured document analysis is appropriate for assessing documented plan comprehensiveness.

Any experiments missing?

No new data collection is required. Additional validation diagnostics and aggregation sensitivity analyses are recommended.

Statistics: Statistical analyses sound?

Partially. Descriptive analyses are appropriate. Validation statistics and treatment of ordinal data require refinement.

Results and data: Support conclusions?

Yes, provided conclusions remain explicitly bounded to documented plan structure.

All data accessible?

Yes. Source documents and scoring datasets are publicly accessible as described.

References: Anything missing?

No critical omissions identified. Consider adding references on rubric based assessment and ordinal agreement interpretation.

Title: Appropriate and informative?

Yes. The title accurately reflects the scope and approach.
