# Supplementary material for: A global analysis of national cardiovascular disease control plans using a multi-agent artificial intelligence model
Source: PLOS Digit Health. 2026 Jun 1;5(6):e0001447. doi: 10.1371/journal.pdig.0001447 (PMC13225395; doi:10.1371/journal.pdig.0001447)
Supplement: Attachment — Submitted filename: PLOS Response to Reviewers_04_05_26_Final.docx [file pdig.0001447.s011.docx]

**Response to Reviewers**

*A global analysis of national cardiovascular disease control plans using a multi-agent artificial intelligence model*

We thank the editor and all three reviewers for their detailed and substantive critiques. The revised manuscript addresses every point raised. Below we respond to each comment in turn, noting specific manuscript changes with line references.

**Editor Comments**

Thank you for the detailed and informative review of our manuscript.

**Comment 1:**

*The reported Pearson correlation between PRISM and blinded human review (r = 0.048) indicates essentially no linear agreement. This critically weakens claims regarding model validity. Moreover, the scoring scale is ordinal (1–5), yet analyses treat it as continuous. The authors must:*

- *Use appropriate statistics for ordinal data (e.g., Spearman correlation, weighted kappa, ICC).*
- *Conduct a formal bias analysis to address the reported +0.28 “generous auditor” effect.*
- *Clarify the interpretation of the scoring scale, including what constitutes a “good” score.*

*Without stronger validation evidence and proper statistical treatment, conclusions about AI reliability are not sufficiently supported.*

**Response:**

Thank you for this important comment regarding model validation, scoring scale and interpretation of findings. We agree with all three concerns.

On appropriate statistics, we have revised the validation analysis to emphasise ordinal-appropriate agreement measures. Pearson's *r* has been removed as a primary measure. We reanalysed the data and report in the Results all N = 403 paired LLM–human observations using ordinal-appropriate statistics: Spearman ρ = 0.061 (p = 0.222), linear-weighted κ = 0.049, quadratic-weighted κ = 0.045, intraclass correlation coefficient (ICC) (3,1) = 0.046 (p = 0.181). We have clarified in the Discussion that these findings indicate similar substantive conclusion — overall LLM–human agreement is weak and non-significant — but on an appropriate statistical footing. A floor effect is present: 61.0% of LLM scores and 62.3% of human scores were 0, inflating per-element exact agreement for elements with sparse policy content. Agreement on elements requiring qualitative judgement was poor (Element 6: 16.7%, Element 7: 19.0%, Element 8: 37.3%); elements requiring structural detection performed markedly better (Element 4: 72.9%; Element 5: 72.9%). (Methods lines 182–209; Discussion lines 384–415)

We have also expanded the bias analysis to stratify by score level. The +0.28 'generous auditor' characterisation was incomplete. Stratified analysis by human score level reveals bidirectional, score-dependent bias: PRISM over-scores when human reviewers assign 0 (mean difference +0.96) but systematically under-scores when reviewers assign ≥2 (mean differences −0.93 to −4.00 at scores 2–5). As each reviewer assessed a non-overlapping subset of countries (Reviewer 1: Ghana and India; Reviewer 2: Myanmar and the United States; Reviewer 3: Turkey and the United Kingdom), no shared observations were available to formally assess inter-rater reliability. The validation therefore relies on three independent but non-overlapping reference standards — a structural limitation acknowledged in the Discussion. This clarification has been incorporated in the validation results and discussed as a limitation of the current workflow (Results lines 193–209; Discussion lines 392–396).

On scoring scale Sub-element scoring criteria were defined prospectively in the analytical framework and applied identically by PRISM and human reviewers. The full rubric is provided in the supplementary materials. Sub-elements were scored on a 0–5 ordinal scale with criteria defined prospectively in the analytical framework: 0 = not discussed; 1 = partially discussed, no recommended indicators used; 2 = partially discussed with some recommended indicators; 3 = fully addressed with some or all recommended indicators; 4 = fully addressed with all recommended indicators and national baseline data; 5 = fully addressed with all indicators, baseline data, and specific measurable targets. A reference to these thresholds has been added to the Methods (lines 119–126).

**Comment 2:**

*Selection Bias and Limited Global Representation*

*The country sample shows substantial imbalance:*

- *22/44 countries are European.*
- *Very few low-income countries are included.*
- *Key global actors are missing.*

*As a result, the global median score is disproportionately influenced by European data. The conclusion that low-income countries perform similarly to high-income countries is statistically unreliable given the extremely small number of low-income cases.

To support global claims, the study must either:*

- *Expand representation of low- and middle-income countries (particularly from Africa, Southeast Asia, and Latin America), or*
- *Substantially temper conclusions and explicitly acknowledge structural sampling bias.*

**Response:**

Thank you for raising this concern. The sampling imbalance is acknowledged. Our sample reflects the universe of accessible, official, non-superseded National CVD plans meeting the prespecified inclusion criteria. Within that sampling frame, the final dataset is the most comprehensive to date and includes 22 of 45 countries that are European (48.9%) and 27 are high-income (60.0%), with Africa (n=4), South-East Asia (n=2), and LIC countries (n=2) under-represented. (We note the total is 45 countries, one more than the 44 cited by the reviewer; this discrepancy arose from a data entry error in the original dataset that has been corrected.) We have revised the description of this structural sampling constraint in the Limitations paragraph of the Discussion (lines 427–433).

Regarding income group comparisons. With n=2 LIC countries, Mann-Whitney pairwise tests are non-significant across all income group comparisons (LIC vs HIC: p=0.709; LMIC vs HIC: p=0.064; UMIC vs HIC: p=0.200). All comparative claims about income group performance — including the 'low-income countries scored similarly to high-income countries' statement — have been removed from the abstract and discussion. Income group results are now presented descriptively only, without inferential conclusions (Abstract lines 42–44; Results lines 309–311; Discussion lines 363–368).

On generalisability. The limitations section has been expanded to name specific structural gaps. Findings are described as most generalisable to HIC, European settings. The scarcity of accessible plans in LIC and African contexts is noted as a substantive finding in its own right. Expanding coverage across Africa, South-East Asia, and Latin America is named as the primary direction for future research (lines 427–433).

**Comment 3:**

*Model Interpretation and Transparency

Given the weak correlation with human scoring, the manuscript must more clearly distinguish between:*

- *Structural identification of policy components, and*
- *Qualitative evaluation of policy depth or feasibility.*

*A concrete example of AI–human scoring disagreement would strengthen transparency.*

*In its current form, the manuscript presents promising methodology but insufficient statistical robustness and limited external validity. These issues must be addressed in a major revision.*

**Response:**

Thank you for this constructive recommendation. We have revised the Results, and Discussion (Results lines 193–209; Discussion lines 384–412) to characterise PRISM’s health policy benchmarking model — validated for assessing comprehensiveness and whether defined policy content is present or absent — and explicitly not validated for assessing quality or implementation feasibility, which must be interpreted within the health system context in which the plan is situated. Per-element agreement data support this: elements requiring structural detection (Elements 4 and 5: exact agreement 72.9% each) show high agreement; elements requiring qualitative judgement (Element 6: 16.7%, Element 7: 19.0%, Element 8: 37.3%) show the worst (Discussion lines 397–399).

To improve transparency, we have also added concrete examples of disagreement between PRISM and blinded human review. Two systematic patterns are described with specific cases. Over-scoring: PRISM assigned scores of 4–5 to Vision (Ghana, USA) and Financing sub-elements (Myanmar) where human reviewers scored 0, rewarding broad strategic language without implementation architecture. Under-scoring: PRISM scored 0 in India where reviewers assigned 3–4 across Governance, Outputs, and Health Services, failing to integrate content across document sections. A supplementary table of all 84 large-disagreement cases (|diff| ≥ 3) is provided (Discussion lines 400–406).

The Discussion has been revised to emphasise both the promise of the PRISM for scalable policy benchmarking in relation to comprehensiveness and its current limitations in external validity and qualitative assessment (Discussion, lines 384–415).

**Reviewer 1 Comments**

Thank you for the useful and comprehensive assessment of our manuscript.

**Comment 1:**

*Interpretation and support of validation results*

*The validation analysis reports moderate exact and near agreement between computational scoring and human review but weak correlation and very low weighted agreement. While these results are transparently reported, their interpretation remains insufficiently developed. As presented, readers may have difficulty understanding the conditions under which the approach is reliable and the specific limitations of the scoring system.*

*Recommendation:*

- *State explicitly, in the Abstract, Results, and Discussion, that the approach is validated for structural benchmarking of documented plan content rather than for fine grained ordinal scoring equivalent to expert judgment.*
- *Provide at least one diagnostic analysis characterizing disagreement structure, such as element level signed differences between computational and human scores or a collapsed low, medium, high score agreement table.*
- *Report human reviewer inter rater agreement if available. If not assessed, this should be stated explicitly and discussed as a limitation of the reference standard.*
- *Clarify whether observed disagreement reflects systematic generosity, score compression, or element specific limitations*

**Response:**

Thank you for this insightful comment. We have expanded the Abstract (lines 45–48), Results, and Discussion to explicitly state that PRISM is validated for policy benchmarking of documented plan comprehensiveness rather than for fine-grained ordinal scoring equivalent to subject-matter expert judgement.

We have expanded the Results to present a disagreement structure by collapsing ratings into low, medium, and high tiers and by describing the main patterns of divergence between PRISM and human review(scores 0–1, n=318: mean difference +0.75; scores 2–3, n=72: −1.22; scores 4–5, n=13: −3.08) (lines 204–207). The Discussion highlights two concurrent failure modes — over-scoring driven by surface-level keyword matching at low scores and score compression across the full range. A supplementary table of all 84 large-disagreement cases (|diff| ≥ 3) is provided (line 406, S8).

Human inter-rater reliability could not be formally assessed: each of the three reviewers covered a non-overlapping pair of countries, yielding no shared observations across reviewers. This is stated in the Results as a structural limitation of the reference standard, with a recommendation for future validation studies to assign at least two independent reviewers to each plan (lines 207–209).

**Comment 2:**

*Treatment of ordinal scores as interval data*

*Sub element scores on a 0 to 5 rubric are analyzed as numeric quantities using Pearson correlation, arithmetic differences, and aggregated summaries. This implicitly assumes interval scale properties that are not justified for rubric based ordinal assessments and complicates interpretation of validation statistics.*

*Recommendation:*

- *Justify the numeric treatment of rubric scores or revise the emphasis of validation analyses toward ordinal appropriate summaries.*
- *Replace or supplement Pearson correlation with rank based or ordinal focused diagnostics.*
- *Clarify that reported numerical differences represent heuristic indicators of directional bias rather than precise quantitative distances.*

**Response:**

Thank you for this methodological clarification. We agree that the 0–5 rubric is ordinal and that the validation analysis should be framed accordingly.

Pearson correlation has been replaced with ordinal-appropriate statistics (Spearman ρ, linear-weighted κ, quadratic-weighted κ, ICC (3,1)) as the primary validation measures. The 0–5 scale is now explicitly identified as ordinal in the Methods (lines 191–197).

Numerical aggregates (medians, IQRs, mean differences) are retained for descriptive purposes — median and IQR are used as primary summary statistics throughout, consistent with ordinal data — but a statement has been added in the Methods to clarify that these represent heuristic indicators of directional patterns rather than precise quantitative distances (lines 222–225).

**Comment 3:**

*Aggregation logic and robustness of conclusions*

*Element level and overall scores are derived from aggregation across multiple sub elements using an implicit equal weighting scheme. The manuscript does not assess whether key findings are robust to reasonable alternative aggregation choices.

Recommendation:*

- *Provide a brief sensitivity analysis demonstrating that the principal conclusions persist under alternative aggregation approaches, such as mean versus median aggregation or equal weighting by element rather than by sub element.*
- *Clarify the rationale for the chosen weighting scheme and define what the total score represents conceptually.*

**Response:**

Thank you for this helpful suggestion. The aggregation rationale is now stated explicitly in the Methods: element and country-level scores are derived by taking the median across constituent sub-elements with equal weight per sub-element, treating breadth of coverage within an element as substantively meaningful. Median rather than mean is the primary summary statistic, consistent with the ordinal scale (lines 226–232).

We have also added a sensitivity analysis comparing median versus mean aggregation and sub-element-equal versus element-equal weighting. The principal findings were unchanged across all approaches. Country-level Spearman correlations between the baseline and each alternative approach were ≥0.91, and regional rank ordering was stable across all methods; these analyses confirm the robustness statement in the manuscript (lines 231–233) and are summarised in Supplementary Table S7.

The results across all four aggregation approaches are summarised below:

| **Method** | **Global Median** | **Global Mean** |
| --- | --- | --- |
| Sub-element-equal, median (baseline) | 1.00 | 0.82 |
| Sub-element-equal, mean | 1.30 | 1.33 |
| Element-equal, mean | 1.18 | 1.22 |
| Element-equal, median | 1.05 | 1.14 |

**Comment 4:**

*Interpretation of regional and income group comparisons*

*The sample is unevenly distributed across regions and income groups, with small counts in several strata. While limitations are acknowledged, some language may still be interpreted as comparative performance assessment rather than descriptive benchmarking of available documents.*

*Recommendation:*

- *Strengthen language throughout to emphasize that regional and income group analyses are descriptive and exploratory.*
- *Avoid phrasing that implies performance ranking or causal interpretation.*
- *Add a brief reminder near stratified results that small subgroup sizes limit precision and generalizability.*

**Response:**

Thank you for this important point. We have revised the manuscript to ensure that regional and income-group analyses are presented as descriptive and exploratory rather than as comparative performance assessments (lines 268–270). A caveat has been added at the opening of the regional results targeting genuinely small subgroups (South-East Asia n=2; Africa and Western Pacific n=4 each); larger groups (Europe n=22, Americas n=8) do not require the same qualification. The Discussion has been revised to note explicitly that Africa and Western Pacific findings are based on n=4 countries and should not be interpreted as representative regional assessments. Income group inferential language was removed in response to the Editor's Comment 2 (lines 362–365).

**Comment 5:**

*Terminology and labeling consistency*

*One table heading retains language related to cancer control rather than cardiovascular disease control and should be corrected.*

**Response:**

Thank you for identifying this error. We have corrected. Table 3 header 'CURRENT HEALTH SYSTEM PERFORMANCE IN RELATION TO CANCER CONTROL' to CVD CONTROL The column previously mislabelled 'Cancer Strategy' in Table 3 has been corrected to 'CVD Strategy' and the element ordering has been aligned with Table 1 (Table 3, lines 263–266). Table 1 has been relabelled for CVD accuracy (Table 1, line 159 area).

**Comment 6:**

*Clarification of score meaning*

*Add a concise statement clarifying that non zero scores reflect documented presence and specificity within plans, not adequacy, effectiveness, or implementation success.*

**Response:**

Thank you for this suggestion. A clarifying statement has been added to the Methods: scores reflect what is written in the plan; they do not indicate whether described policies are adequate, effective, or implemented (lines 218–222).

**Comment 7:**

*Reproducibility information*

*Provide minimal computational reproducibility metadata, including model version identifiers and the availability of scoring schemas, prompts, or structured output formats.*

**Response:**

Thank you for this recommendation. Model version identifiers are already reported in the Methods (document ingestion: Qwen2.5-VL-72B; policy analysis: Llama 4 Scout 70B). The pipeline architecture is described in S1. A Data Availability statement has been added confirming that the analytical framework and scoring rubric are provided in the supplementary materials, and that structured prompt templates and JSON output schemas are available from the corresponding author on reasonable request (lines 516–519).

**Comment 8:**

*Multilingual and document heterogeneity*

*Briefly describe whether scoring reliability varied by language or document format and how ingestion and validation steps addressed this variability.*

**Response:**

Thank you for this helpful comment. We clarify in the Methods that the document ingestion agent (Qwen2.5-VL-72B) was selected for its multilingual capability and robustness to heterogeneous document layouts, applying spatial-aware text extraction and visual recognition to handle variation in format, structure, and script. Plans were included in any language per protocol. Formal analysis of scoring reliability by language or document format was not conducted; this has been added to the limitations (lines 420–426).

**Comment 9:**

*Contextual elements scoring criteria*

*Clarify whether narrative contextual discussion without explicit headings was eligible for scoring and how implicit content was handled, given the very low scores for threats and opportunities.*

**Response:**

Thank you for raising this point. The scoring rubric defines a score of 1 as content being 'discussed' — narrative contextual discussion without explicit headings is sufficient to score above zero. A clarifying statement has been added to the Methods (lines 174–176). The very low observed scores for threats (median 0.12) and opportunities (0.29) are acknowledged; RAG-based retrieval may perform less reliably on contextual content dispersed across narrative text than on explicitly structured sections, and this has been added to the limitations (lines 421–423).

**Comment 10:**

*The validated framework represents a substantial contribution and should be emphasized as a reusable policy instrument independent of the computational workflow. The manuscript is strongest when it positions the computational system as a scalable tool for structural benchmarking and prioritization for expert review, rather than as a substitute for qualitative policy assessment.*

**Response:**

Thank you for this thoughtful observation. Agreed. The framing of PRISM as augmenting rather than replacing expert review is established throughout the revised manuscript. A sentence emphasising the validated framework as a standalone reusable instrument has been added to the opening of the Discussion (lines 342–343).

**Reviewer 2 Comments**

Thank you for your thorough and helpful review of the manuscript. **Comment 1:**

*The final scoring scale of 1 to 5, it's unclear what a "good" score is of what these points mean. the highest scores in strategy, 2.8, being a bit over half seemed bad to me, but this discussion notes this result as "successful" (line 309). Some clarify about what the score means and what is "good" would be helpful to interpret the results.*

**Response:**

Thank you for this important comment. This comment is substantially addressed by revisions made in response to Editor Comment 1, which outlines the expanded clarifications added to the Methods, Results, Discussion and Supplementary text. The full 0–5 scoring rubric is provided in the supplementary analytical framework (S5), and its criteria are now stated explicitly in the Methods (lines 119–126): a score of 0 indicates content is not discussed, scores of 1–2 indicate partial discussion with limited or no recommended indicators, and scores of 3–5 indicate progressively fuller coverage with recommended indicators, baseline data, and measurable targets. A median strategy score of 2.80 therefore falls short of full substantive coverage. Interpretive language in the Results that mischaracterised scores without scale reference has been corrected: 'low-to-moderate degree of completeness' (overall median 1.20) has been revised to reflect that most plans address framework elements vaguely or only partially, and 'moderate coverage' applied to elements scoring 1.44–1.75 has been corrected to reflect insufficient specificity for substantive coverage (lines 253–255, 261–262).

**Comment 2:**

*Second, there seems to be some formatting errors in Table 3. The median score of 2.8 is for element 6 "threats" but the results text says this score is for strategy, also the table "Cancer strategy" not "CVD". and the order of the elements in Table 1 and Table 3 are not consistent.*

**Response:**

Thank you for identifying these table inconsistencies. We have carefully corrected the residual formatting and labelling errors in Table 3, which have been discussed under comment 5 for reviewer 1 (lines 263–266).

**Reviewer 3 Comments**

Thank you for the detailed and informative review of our manuscript.

**Comment 1:**

*The study’s sample may have some selection bias. Specifically, the number of low-income countries included is very small, and the regional distribution is uneven—with 22 countries in Europe, but only 2 in Southeast Asia and 4 in Africa. This means the global median score is heavily skewed by the European data. Furthermore, data from key representative countries like China and the US is missing. Drawing the conclusion that "low-income countries perform similarly to high-income ones" based on only two data points is statistically unreliable and could be misleading.*

**Response:**

Thank you for noting this. As noted in our previous response to Editor Comment 2, all inferential income group conclusions have been removed; LIC n=2 is explicitly insufficient for any comparison. The limitations section has been expanded to name Africa, South-East Asia, and LIC contexts as substantially under-represented, and findings are reframed as most generalisable to high-income, European settings (lines 427–433).

The United States is included in the sample. China does not have an accessible, official, standalone national CVD control plan meeting our inclusion criteria — broader NCD plans with CVD components were excluded per protocol. This is now noted in the limitations (line 431).

On European overrepresentation: contrary to the concern that European data skews the global median upward, Europe's median total score (1.14) is lower than the Americas (1.26), Eastern Mediterranean (1.46), and Western Pacific (1.71). European overrepresentation does not inflate the global figure.

**Comment 2:**

*The study shows that the Pearson correlation between PRISM and human blind reviews is only r=0.048. In statistics, this is essentially the same as having no linear correlation. Additionally, the authors mention a "generous auditor" effect, where the AI scores are an average of 0.28 points higher than human scores. It is worth exploring whether this systematic bias can be fixed by using "few-shot prompting" or by introducing stricter rules for negative evaluations.*

**Response:**

Thank you for this valuable suggestion. Pearson r has been replaced with ordinal-appropriate statistics (Spearman ρ = 0.061, linear-weighted κ = 0.049, quadratic-weighted κ = 0.045, ICC = 0.046), and the bias has been recharacterized as bidirectional and score-dependent rather than a simple upward shift (lines 191–197).

Few-shot prompting and stricter negative evaluation criteria are well-established approaches to reducing LLM scoring leniency. A sentence naming these as the primary directions for prompt engineering refinement in future iterations of PRISM has been added to the Discussion (lines 413–415).

**Comment 3:**

*There is also the question of a "keyword-trigger" effect. Does the AI give high scores simply because it sees certain words? In contrast, human experts evaluate the logical depth and feasibility of the content. To illustrate this, the paper should include an extreme comparison—specifically, a case where the AI gives a high score while a human reviewer gives a low one.*

**Response:**

Thank you for this important point. We agree that both the potential keyword-trigger effect warrants explanation. The keyword-trigger effect is now explicitly named and mechanistically described in the Discussion (lines 395–396). Cases where PRISM assigned scores of 4–5 while human reviewers scored 0 — in Vision (Ghana, USA) and Financing sub-elements (Myanmar) — involve broad aspirational language without implementation architecture, consistent with the model rewarding stated intent over demonstrated specificity. The contrasting under-scoring pattern in India illustrates the complementary failure: PRISM scored 0 where reviewers assigned 3–4 across Governance, Outputs, and Health Services, failing to integrate content across document sections (lines 400–406). A supplementary table of all 84 large-disagreement cases (|diff| ≥ 3) is provided (line 406).

**Comment 4:**

*The research framework is adapted from a 2008 cancer control plan, even though CVD and cancer have very different management models. While the paper mentions that the framework was updated using the Delphi method, Table 1 still seems to show traces of the original version. For example, Element 3 includes "Cancer Surveillance Systems." Please verify if the use of the word "cancer" here is simply a typo.*

**Response:**

Thank you for pointing out this error. The framework was comprehensively recontextualised for CVD through literature review, guidance from the World Heart Federation and World Health Organisation, and an extensive consultatory two-stage Delphi process involving 42 specialists as described in the Methods — no cancer-specific content was retained. The residual cancer terminology in both tables is a transcription error (Table 1, line 159, Table 3, lines 263–266).

**Comment 5:**

*While this study shows the huge potential of AI for large-scale policy analysis, its effectiveness as a qualitative tool should still be interpreted with caution.*

**Response:**

Thank you for this balanced assessment.We tend to agree and have revised the manuscript to make this caution explicit. . PRISM's validated scope is defined in the Methods as structural identification of policy architecture presence. The Discussion explicitly states that PRISM does not replicate expert qualitative depth assessment, supported by ordinal agreement statistics and per-element breakdown. The limitations paragraph concludes that conclusions about policy depth, implementation readiness, or strategic sophistication should not be drawn from PRISM scores without independent expert review (lines 434–435).
